# Lafora disease E3-ubiquitin ligase malin is related to TRIM32 at both the phylogenetic and functional level

**DOI:** 10.1186/1471-2148-11-225

**Published:** 2011-07-28

**Authors:** Carlos Romá-Mateo, Daniel Moreno, Santiago Vernia, Teresa Rubio, Travis M Bridges, Matthew S Gentry, Pascual Sanz

**Affiliations:** 1 Instituto de Biomedicina de Valencia, CSIC and Centro de Investigación Biomédica en Red de Enfermedades Raras (CIBERER), Jaime Roig 11, 46010-Valencia, Spain; 2 Department of Molecular and Cellular Biochemistry and Center for Structural Biology, University of Kentucky, Lexington, KY 40536-0509, USA

**Keywords:** AMPK, malin, TRIM32, E3 ubiquitin ligase, phylogeny, Lafora disease

## Abstract

**Background:**

Malin is an E3-ubiquitin ligase that is mutated in Lafora disease, a fatal form of progressive myoclonus epilepsy. In order to perform its function, malin forms a functional complex with laforin, a glucan phosphatase that facilitates targeting of malin to its corresponding substrates. While laforin phylogeny has been studied, there are no data on the evolutionary lineage of malin.

**Results:**

After an extensive search for malin orthologs, we found that malin is present in all vertebrate species and a cephalochordate, in contrast with the broader species distribution previously reported for laforin. These data suggest that in addition to forming a functional complex, laforin and perhaps malin may also have independent functions. In addition, we found that malin shares significant identity with the E3-ubiquitin ligase TRIM32, which belongs to the tripartite-motif containing family of proteins. We present experimental evidence that both malin and TRIM32 share some substrates for ubiquitination, although they produce ubiquitin chains with different topologies. However, TRIM32-specific substrates were not reciprocally ubiquitinated by the laforin-malin complex.

**Conclusions:**

We found that malin and laforin are not conserved in the same genomes. In addition, we found that malin shares significant identity with the E3-ubiquitin ligase TRIM32. The latter result suggests a common origin for malin and TRIM32 and provides insights into possible functional relationships between both proteins.

## Background

Protein regulation by ubiquitination is a highly conserved process in eukaryotic organisms. Ubiquitin and ubiquitin-like molecules label proteins for either protein degradation or signalling purposes, and deficiency in the ubiquitination machinery leads to severe pathologies [1]. Ubiquitination takes place by means of the coordinated action of three different kinds of enzymes: E1s (ubiquitin-activating enzymes), E2s (ubiquitin-conjugating enzymes) and E3s (ubiquitin ligases). Among all these proteins, the most diverse are the E3-ubiquitin ligases, which are counted by hundreds in humans and confer high specificity to the process [1]. This group is further divided into three subfamilies, HECT-type, RING-type E3-ubiquitin ligases, and U-box ligases. The RING-type family of E3-ubiquitin ligases constitutes a diverse group of enzymes, and in vertebrate organisms, combination of accessory domains following the RING domain leads to highly specific variants that facilitate protein-protein interactions [2]. An example of the functional diversity accomplished by combination of RING and other domains can be found in the large family of enzymes known as TRIM proteins. TRIM (TRIpartite Motif-containing) proteins are characterized by a common core structure (a RING domain, one or two B-box domains, and a coiled-coil region) and accessory domains that make this family diverse at both structural and functional levels [3]. TRIM proteins have so far been involved in processes that include cell proliferation, differentiation, development, oncogenesis and apoptosis, and some of them have also been shown to display antiviral properties ([4-6]).

Malin is a RING-type E3 ubiquitin ligase with six NHL domains [a protein domain present in Ncl-1, HT2A (TRIM32) and Lin-41 proteins] [7] located in its C-terminus [8]. NHL domains are usually involved in protein-protein interactions. Malin forms a functional complex with the glucan phosphatase laforin, and this interaction is dependent on the NHL domains in malin. In this complex, laforin directs malin towards physiological substrates ([9-11]). Thus, laforin is a targeting subunit for malin or a scaffold that brings together substrate and E3 ligase. Mutations in the genes encoding malin (*EPM2B*) or laforin (*EPM2A*) lead to the development of Lafora progressive myoclonus epilepsy ([12-14]), and patients with mutations in either one of these genes show similar pathological presentations. Malin depends on laforin interaction to perform its physiological function as an E3-ubiquitin ligase, ubiquitinating substrates involved in glycogen metabolism such as glycogen synthase and PP1 regulatory subunit R5/PTG. While multiple groups have corroborated these findings using different systems, deletion of the malin gene in a mouse model does not result in increased accumulation of these proteins [15]. Therefore, these incompatible results are currently under investigation.

Impairment of the laforin-malin functional complex results in deregulation of glycogen metabolism and formation of insoluble glucans [10]. The interaction between malin and laforin is modulated by AMP-activated protein kinase (AMPK) [10], and conversely, we have recently described the ubiquitination of AMPK subunits by the laforin-malin complex [16]. Since AMPK also phosphorylates R5/PTG and this phosphorylation increases the ubiquitination and further degradation of this protein by the laforin-malin complex [17], there must exist a functional link between laforin, malin, AMPK and R5/PTG that results in the control and regulation of glycogen synthesis.

Laforin and malin belong to structurally and functionally different protein families, yet they function, in mammals, as a tightly linked functional complex. This concept gives rise to the question whether laforin and malin could have additional independent cellular functions. If these independent functions exist, it should be possible to find organisms with laforin or malin and without the second component of the complex. We recently defined the evolutionary lineage of the laforin gene [18-20]. Although it was previously assumed that the gene encoding laforin (*EPM2A*) was a vertebrate-specific gene, we identified several laforin orthologs in distant organisms, including invertebrates and protists. In addition, we identified a laforin-like protein in all organisms of green algal descent. However, no data on malin or similar proteins have been reported in non-vertebrate organisms, thus, the physiological functions of malin independent of those coordinated with laforin remain unclear.

In this work, we performed a phylogenetic study of malin that indicates malin is present in all vertebrate species and a cephalochordate. Importantly, this pattern of species distribution does not correlate with the species distribution of laforin, which suggests that laforin and/or malin may have additional independent functions. In addition, we found that malin shares significant identity with the E3-ubiquitin ligase TRIM32, and it is phylogenetically related to the TRIM family of proteins. The commonalities between malin and TRIM32 are not reserved to just phylogenetic relationships. We demonstrate that TRIM32 can ubiquitinate malin targets in tissue culture cells, although the type of ubiquitin chain that TRIM32 incorporates into these substrates is different than malin. On the contrary, malin cannot ubiquitinate TRIM32 substrates. Cumulatively, these data define the evolutionary lineage of malin, provide evidence that laforin likely possesses malin-independent functions, and uncovers structural and functional similarities between malin and TRIM32.

## Results

### 1. Malin is conserved in all classes of vertebrates

We recently defined the evolutionary lineage of the laforin gene [18-20]. The laforin gene is conserved in all vertebrate genomes, but it is absent in genomes of most non-vertebrate organisms including the standard model organisms yeast, flies, or worms. Surprisingly, we found that laforin is conserved in the cephalochordate *Branchiostoma floridae *and in the Cnidarian *Nematostella vectensis *as well as in the following five protozoans Cyanidioschyzon *merolae, Toxoplasma gondii, Eimeria tenella, Tetrahymena thermophila*, and *Plasmodium tetraurelia *[18,19]. Thus, laforin possesses an ancient and unique evolutionary lineage. Given that the function of laforin and malin has been linked in vertebrates, we sought to determine if this was true in all organisms. If they are not conserved in the same species, then these data would argue for malin- and/or laforin-independent functions.

**Figure 1:**
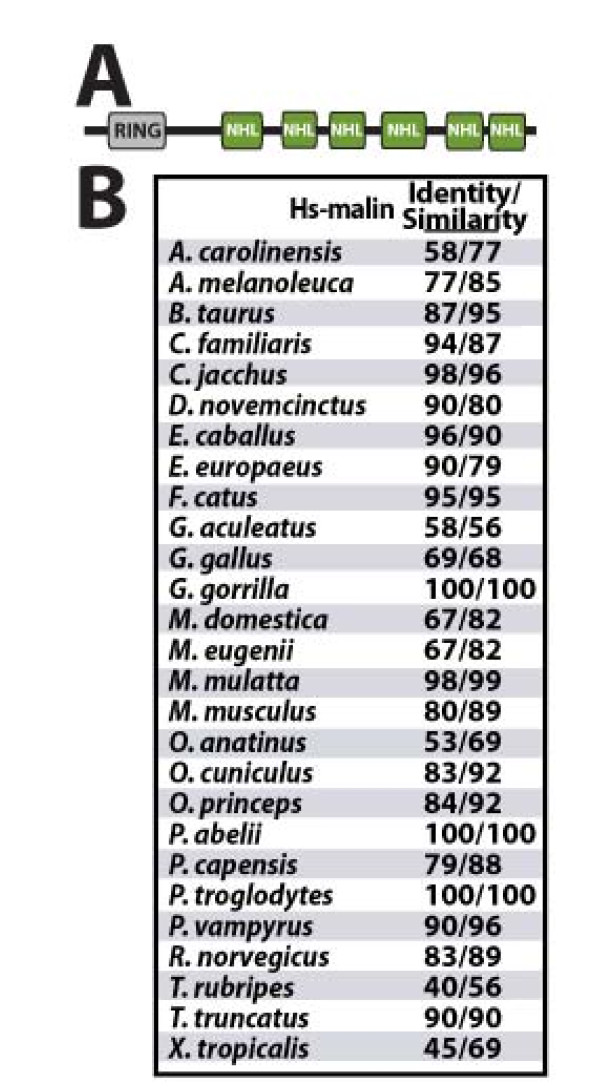
**Conservation of malin in vertebrate genomes**. A) Schematic depicting the domains present in malin. B) Protein sequences from all vertebrate malin orthologs were utilized to generate an alignment (additional file 1, Figure S1). This alignment was used to compare the percent similarity and identity of each malin ortholog to human malin.

Malin is a RING-type E3 ubiquitin ligase and contains six NHL domains (Figure 1A). NHL domains form a six-bladed β-propeller and direct protein-protein interactions, similar to WD40 domains [7,21]. In order to define the evolutionary lineage of malin, we first searched vertebrate databases using the criteria that a malin ortholog must contain a RING domain and six NHL domains, in any orientation (i.e. amino- versus carboxy-terminal), and that it cannot contain any other domains. We utilized the NCBI non-redundant (nr) protein database, organism-specific databases, and the SUPERFAMILY database [22,23]. We performed BLASTp and PSI-BLAST searches of these databases and considered any protein with an E value < 1e-90 a probable malin ortholog and proteins with < 1e-50 a possible ortholog. We analyzed each of these sequences using PROSITE, PFAM, JPRED3, and CDD [24,25] to define predicted secondary structure as well as determine if additional domains were present and excluded proteins with any additional domains.

We identified 28 vertebrate genomes containing a protein with a RING domain and six NHL domains (Figure 1B and additional file 1, Figure S1). For each genome that contains a malin ortholog, we found that malin was the only protein in the genome that contained only these two domains. Each malin ortholog identified had an E value < 1e-90, contained a RING domain and six NHL domains with similar boundaries as human malin, and possessed similar predicted secondary structure. We searched each of the malin orthologs for additional domains, but did not identify other putative domain in the malin orthologs. To ensure that we had identified malin orthologs, we generated a MAFFT [26] alignment of the 28 proteins to determine their percent identity and similarity to *H. sapiens *malin (Hs-malin) as well as compared predicted secondary structure. The similarities ranged from 40% for Actinopterygii (fish) versions of malin to 100% for other primate versions of malin (Figure 1B). Of note, we identified a malin ortholog in at least one member of each class of vertebrates. Thus, malin is conserved in all five classes of vertebrates, and it is the only protein in vertebrate genomes that contains only a RING and six NHL domains.

### 2. Malin is conserved in all vertebrate genomes and one cephalochordata genome

In addition to vertebrate genomes, we also probed the NCBI databases (BLASTP) for a malin ortholog in invertebrate and protozoan genomes. We did not uncover a malin ortholog in any non-vertebrate genome NCBI database. Given the lack of non-vertebrate sequences obtained in our BLASTP searches, we wondered whether the malin gene was absent in other taxa or not observed due to incomplete sequencing data.

**Figure 2:**
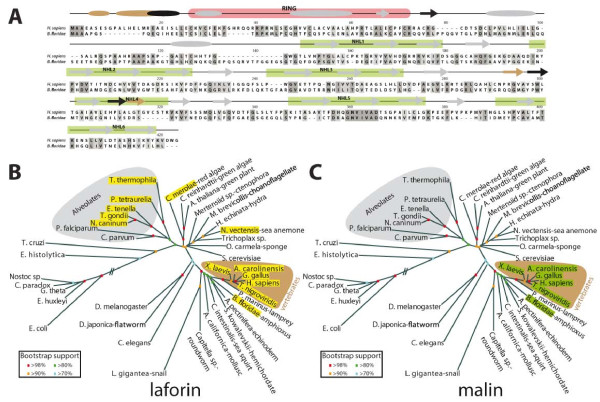
**Malin and laforin orthologs**. A) *H. sapiens *and *B. floridae *malin were aligned using MAFFT and displayed in MacVector. Predicted secondary structure is displayed over the sequences with ovals representing α-helices and arrows representing β-sheets. Grey structural elements are shared by *H. sapiens *and *B. floridae *malin, black domains are specific to *B. floridae *malin, and tan domains are specific to *H. sapiens *malin. The RING domain is highlighted in light red and the NHL domains are highlighted in light green. B) Unrooted phylogeny of the small subunit ribosomal RNA (SSU rRNA) sequences was generated as described in Methods. Organisms containing laforin are boxed in yellow. Alveolates are shaded with a gray background and vertebrates with a brown background. Bootstrap values are indicated by color-coding according to the insert. C) Unrooted phylogeny of SSU rRNA as in *B*), but organisms containing malin are boxed in green.

To answer these questions, the same queries were used to perform BLASTP, TBLASTN, PSI-BLAST, PHI-BLAST, and domain searches against species-specific databases. We probed 215 species-specific databases from *Bilateria, Cnidaria, Ctenophora, Placozoa, Porifera, Choanoflagellate*, as well as single-celled protozoan genomes, and 1,408 bacterial genomes (additional file 2, Table S1). Only sequences including similarity at both RING and NHL domains were considered as positive hits. To increase the breadth of our search, data from ENSEMBL gene database [27] were also investigated. These efforts identified a malin ortholog in one taxon outside of vertebrates. We identified a malin ortholog in the cephalochordate *Branchiostoma floridae *(Figure 2A). Although we closely examined the genomes of all metazoans, protists, plants and bacteria, we did not identify a malin ortholog in any of these other genomes. We did identify proteins with either a RING domain or NHL domains in genomes as ancient as archae (additional file 3, Figure S2 and additional file 4, Figure S3). However, these proteins only contain a RING or NHL domains, and none of them contain both domains. Thus, RING and NHL domains are found independently in the most basal genomes, but they are not observed within the same protein until the emergence of malin.

One of the reasons for performing these analyses was to determine if malin shared a similar evolutionary lineage with laforin. Laforin is a glucan phosphatase that contains a carbohydrate binding module (CBM) and dual-specificity phosphatase (DSP) domain, and physically interacts with malin to form a functional complex. When we compared the species distribution of malin with that previously described for laforin [18,19], we observed that laforin and malin do not correlate in species distribution (Figure 2B &2C). Since laforin is present in the genome of more evolutionarily basal organisms than malin, these results suggest that laforin may perform additional functions independent of malin. It is possible that these functions are conserved from red algae to humans, but our results indicate that at least in lower eukaryotes laforin must posses malin-independent functions.

### 3. Malin is phylogenetically related to the TRIM family of proteins

While malin is the only protein that we found in vertebrate genomes containing only a RING and six NHL domains, our bioinformatic analyses did recover other proteins with RING and NHL domains. The protein with the highest identity to malin that we recovered corresponds to TRIM32, a E3-ubiquitin ligase that belongs to the tripartite-motif containing family of proteins [3]. TRIM32 is characterized by the presence of the conserved TRIM core domains (a RING, B-box, and coiled coil) and also by the presence of several NHL domains, which resemble the NHL domains of malin.

**Figure 3:**
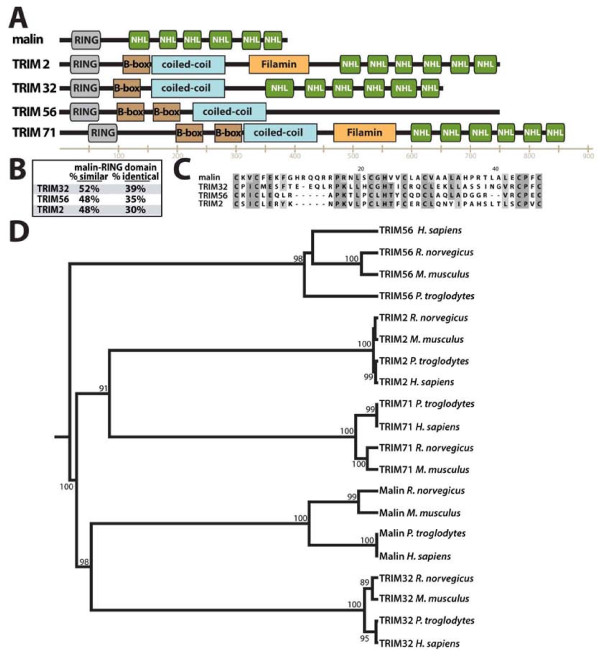
**Similarities between malin and the TRIM family of proteins**. A) Schematic depicting the domains present in malin, TRIM2, TRIM32, TRIM56, and TRIM71. All domains and domain placement are to scale, and based on analyses using PROSITE and PFAM. B) The RING domain of malin, TRIM2, TRIM32, and TRIM56 were aligned and analysed for percent similarity and identity. C) CLUSTALX alignment of the RING domains of malin, TRIM2, TRIM32, and TRIM56. D) Phylogenetic relationships between malin and TRIM proteins in mammals. Sequences of rat (*R. norvegicus*), mouse (*M. musculus*), human (*H. sapiens*) and chimp (*P. troglodytes*) orthologs for malin and related TRIM proteins, obtained after BLASTP searches, were aligned using MAFFT (additional file 5, Figure S4) and used for the construction of a maximum-likelihood phylogenetic tree as described in Material and Methods.

Given the similarity between malin and TRIM32 we decided to further investigate the phylogenetic relations between malin and TRIM proteins so there would be no confusion between TRIM and malin orthologs. Sardiello et al [28] recently defined the TRIM protein family, and classified them into Group 1 and Group 2 based on relatedness and their rates of evolution. Malin is more similar to the 34 TRIM proteins in Group 1, some of which contain NHL domains. We chose four members from Group 1 to further analyze and compare/contrast with malin. TRIM2 and TRIM32 each contain a RING, B-box, coiled-coil, and six NHL domains, and TRIM2 also contains a Filamin domain (Figure 3A). TRIM 56 and TRIM71 each contains different combinations of these five domains (Figure 3A). First, we analyzed the RING domains of TRIM2, TRIM32, and TRIM56, and found that the TRIM32 RING shares the highest degree of similarity and identity with the malin RING (Figure 3B and 3C). In fact, when we performed a BLASTP search with the human malin RING domain in the NCBI *H. sapiens *nr database, the RING domain from TRIM32 is the first non-malin hit.

Next, we performed BLASTP searches with human TRIM2, TRIM32, TRIM56, and TRIM71, with the aim of unveiling the phylogenetic relations between malin and this group of TRIM proteins. We verified orthologs of each protein by analyzing their domain boundaries and domain arrangement, and generated an alignment from the sequences of malin, TRIM2, TRIM32, TRIM56, and TRIM71 from *H. sapiens, P. troglodytes, R. norvegicus*, and *M. musculus *(additional file 5, Figure S4). Using the alignment, we generated a phylogenetic maximum-likelihood tree of mouse, rat, chimp and human orthologs (Figure 3D). The tree confirms that malin and TRIM32 orthologs are the closest homologs with TRIM71, TRIM56, and TRIM2 orthologs as more divergent from malin.

To gain insight into the evolution of the malin and TRIM orthologs, we analyzed the gene structure and intron-exon boundaries of each. The gene encoding malin (*EPM2B*) is a single exon in all mammalian, bird, fish, and amphibian genomes (27 genomes in total), but the malin gene contains two exons in reptile genome (additional file 6, Figure S5). The *B. floridae *malin ortholog is also a single exon. The genes encoding TRIM2 and TRIM71 both contain multiple exons (3-11 exons) in all genomes investigated, including: mammals, bird, fish, amphibian, reptiles, *B. floridae, C. elegans, C. intestinalis, D. melanogaster*, and *N. vectensis *(additional file 6, Figure S5). Therefore, it seems unlikely that the malin gene is most closely related to either TRIM2 or TRIM71. Alternatively, TRIM32 and TRIM56 are both single exon genes in all mammalian genomes. We were only able to identify TRIM56 in mammalian genomes, but it is a single exon gene in all twelve genomes where we identified it. Similarly, TRIM32 is a single exon gene in all fourteen mammalian genomes where it was identified, and in the two amphibian genomes. However the TRIM32 gene contains two exons in bird, fish, and reptile genomes (additional file 6, Figure S5). Due to the limited sequence data available for TRIM56, it is difficult to definitively determine if malin is more similar at the gene structural level to TRIM32 or TRIM56. However, TRIM56 does not contain NHL repeats in its sequence while TRIM32 does contain NHL domains and malin and TRIM32 share many similarities even at the gene level. Thus it seems likely that malin is more similar to TRIM32 at both the protein and gene level.

**Figure 4:**
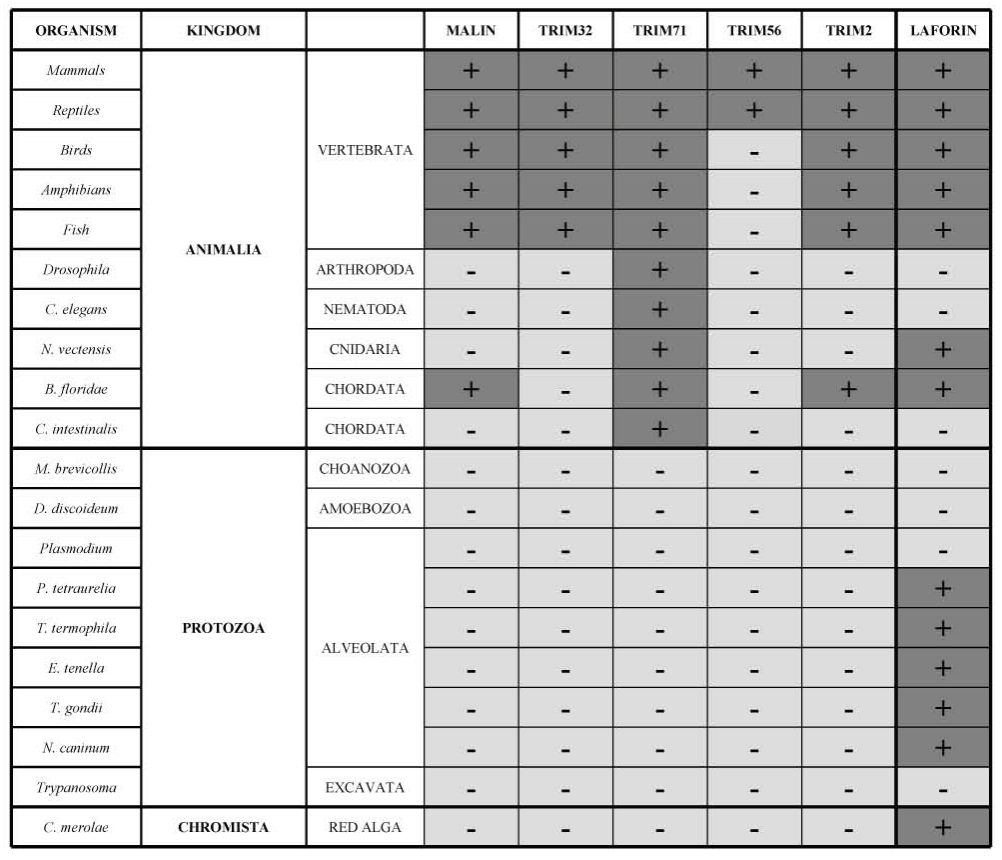
**Malin and TRIM32 are present in vertebrate species**. Summary of the results obtained by BLASTP searches and phylogenetic tree constructions. Analyzed species are shown in the left column (in some cases, a whole genus was submitted to BLASTP searches, i.e. *Drosophila *and *Trypanosoma*). Species are ordered by kingdoms and taxonomic groups following classification as in [43]. Proteins found in those organisms are marked by a (+) sign (dark grey); undetected proteins are marked as (-) (light grey). Malin and TRIM32 were found in vertebrate species and malin is also present in one cephalochordata genome. Neither of the TRIM proteins analyzed, nor malin, were present in organisms belonging to kingdoms protozoa or chromista, in contrast with the species distribution found for laforin (right column). For a detailed list of protein entries, see additional file 7, Table S2.

Regarding species representation of TRIM proteins, our analyses confirm previous reports on TRIM family conservation [28]. Although all TRIM proteins have several mammalian orthologs (Figure 4), there are some exceptions that are likely due to incomplete genome sequencing or gene loss: TRIM32 is absent in *Gallus gallus *and *Xenopus laevis *(a TRIM32 bird ortholog annotated in ENSEMBL is more similar to TRIM2). TRIM56, present in all mammalian genomes, is absent in monotremes, aves, amphibians and fish. TRIM71 and TRIM2 are clearly the most extended proteins from the analysed group. However, sequence divergence makes it difficult to define the correct orthology between *Nematostella vecten*sis sequences similar to TRIM2 and TRIM71. In addition, TRIM71 is uniquely conserved in nematodes and insects. However, no significant identity with malin or any of these four TRIM proteins was found in protozoan (Figure 4) or fungal genomes (not shown). Cumulatively, these data define the differences between the TRIM family and the RING-NHL protein malin.

### 4. Malin and TRIM32 share sequence and structural features

**Figure 5:**
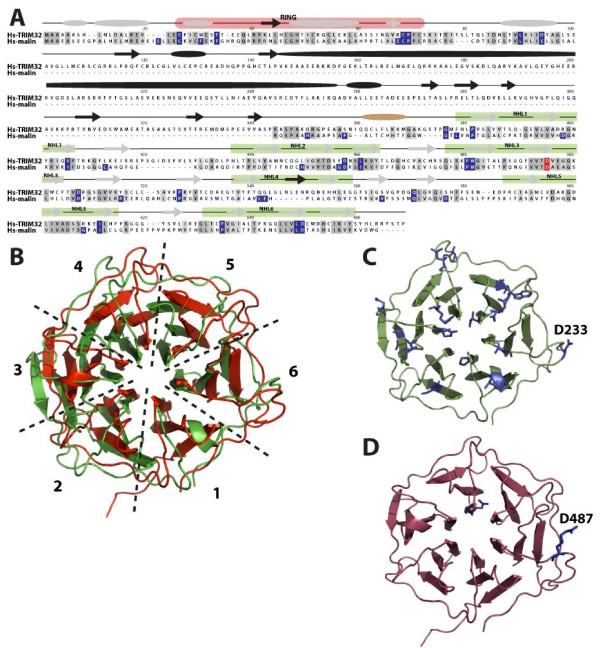
**Domain organization and sequence alignment of malin and TRIM32**. A) Malin shares sequence features with TRIM32. Human sequences were aligned using MAFFT and displayed in MacVector. Predicted secondary structure is displayed over the sequences with ovals representing α-helices and arrows representing β-sheets. Grey structural elements are shared by malin and TRIM32, black domains are specific to TRIM32, and tan domains are specific to malin. The RING domain is highlighted in light red and the NHL domains are highlighted in light green. Malin-specific Lafora disease missense mutations are highlighted in blue and when conserved the similar amino acid in TRIM32 is highlighted in blue. The position of D233 in malin and D487 in TRIM32 is highlighted in red. B) Alignment of malin and TRIM32 structural models. NHL-containing fragments of both sequences (amino acids 113-396 in malin; 358-646 in TRIM32) were submitted to the ESypred3D server and modeled using structure of *M. tuberculosis *PknD (PDB:1rwl) as a template. Both structural models (green for malin, red for TRIM32) were displayed and aligned using PyMOL, showing a repetition of six NHL domains in both cases. C) Mapping of mutations found in patients in structural models of malin. Amino acids reported to cause disease are shown in blue. D) Mapping of Limb-Girdle muscular dystrophy mutations in TRIM32. The position of D487N is highlighted and is structurally analogous to malin-D233A Lafora disease mutation.

Given the similarities between malin and TRIM32, we decided to further investigate these two proteins. An alignment of human TRIM32 and malin illustrates a high degree of identity at both the N-terminus (corresponding with the RING domain in both proteins) and the C-terminus (corresponding to the NHL repeats) (Figure 5A). Malin and TRIM32 are 27% similar overall, and 52% and 38% similar between their RING and NHL domains, respectively. However, TRIM32 possesses a portion that spans from amino acids 198 to 285 (between the B-box domain and the first NHL domain) that is absent in malin. We then mapped mutations in the malin gene found in Lafora disease patients onto the protein alignment (Figure 5A, highlighted in blue). Twenty-one of the thirty-seven Lafora disease missense mutations in malin contained a conserved amino acid in TRIM32. One of those conserved residues (D233 in malin, highlighted in red) aligns with D487 in TRIM32. Interestingly, this amino acid is mutated to malin-D233A in Lafora disease patients [29] and TRIM32-D487N in Limb-Girdle muscular dystrophy patients [30].

To further asses these observations, we generated structural models for the NHL domains of both proteins using the crystallised NHL domain of *M. tuberculosis *PknD (PDB:1rwl [31]) as a template, which has an identity of 23.5% with malin and 22% with TRIM32. An alignment of the predicted structures (Figure 5B) shows that both the malin and TRIM32 models contain six repeats of the characteristic three/four β-sheet found in NHL domains [21]. Malin mutations analysed in Figure 5A locate mainly in segments of the structure corresponding to β-sheets (Figure 5C). Given their location, it seems probable that deleterious mutations confer structural issues in the NHL domains and likely result in non-functional protein-protein interaction modules. It was especially interesting that residues D233 in malin and D487 in TRIM32 are located in equivalent positions not only at primary structure (Figure 5A), but also at predicted three-dimensional structure level (Figure 5C and 5D). The similarity of these two E3 ubiquitin ligases and their overall conservation for pathologically relevant amino acids prompted us to compare both proteins at a functional level.

### 5. Malin and TRIM32 are related at functional level

**Figure 6:**
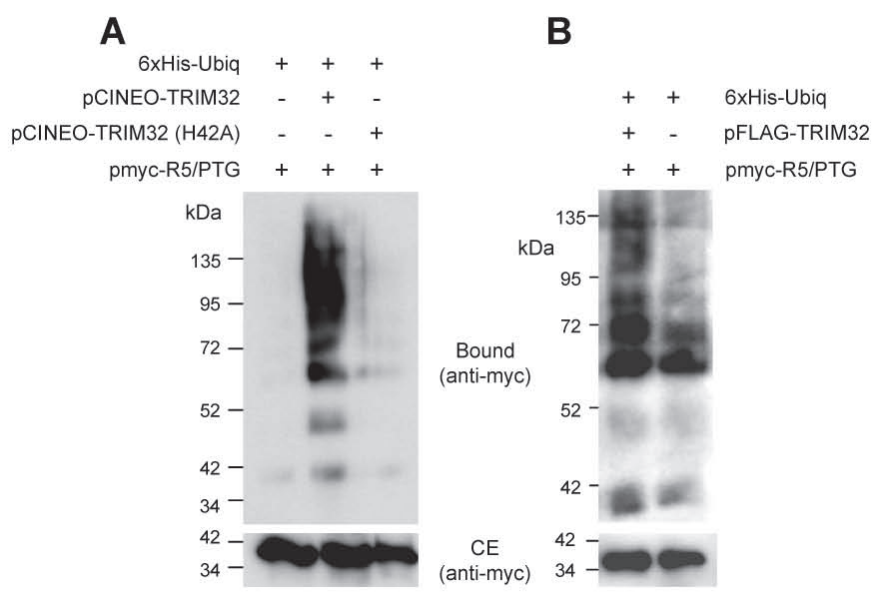
**TRIM32 ubiquitinates recognized malin substrates such as R5/PTG**. A) HEK293 cells were co-transfected with 6xHis-tagged ubiquitin constructs, myc-R5/PTG, and either pCINEO-TRIM32, pCINEO-TRIM32 (H42A) (encoding a catalytically inactive form of TRIM32) or empty vector. Cell extracts were obtained as described in Materials and Methods, and samples from crude extracts (CE, 30 μg) and material bound to the metal affinity column (Bound) were analyzed by SDS-PAGE and Western blotting using anti-myc antibodies, as described in Materials and Methods. B) MEF cells from epm2b-/- mice lacking malin were co-transfected with 6xHis-tagged ubiquitin constructs, myc-R5/PTG and pFLAG-TRIM32 or empty plasmids. Cell extracts were analyzed as in part A.

In order to determine whether malin and TRIM32 could have redundant functions, we first studied a physiological substrate of malin that is related to alterations found in Lafora disease. The PP1 regulatory subunit R5/PTG is ubiquitinated by the laforin-malin complex and labelled for degradation [9,17]. Thus, we analysed whether TRIM32 was able to ubiquitinate R5/PTG. With this aim, we transfected HEK293 cells with His-tagged ubiquitin constructs, myc-R5/PTG and pCINEO-TRIM32 and purified ubiquitinated proteins by metal-affinity column purification [16,32]. R5/PTG was only ubiquitinated in cells transfected with TRIM32 (Figure 6A). In order to analyze the specificity of the reaction, the same assay was conducted with a catalytically inactive form of TRIM32 with a H42A mutation in the RING domain (TRIM32 H42A). In this case no ubiquitination of R5/PTG was observed (Figure 6A). In order to discard the possibility that the action of TRIM32 on R5/PTG was mediated by endogenous malin, we repeated the ubiquitination experiments in mouse embryonic fibroblast (MEF) cells from a mouse model lacking malin (epm2b-/- mouse). As observed in Figure 6B, the expression of TRIM32 in these cells still promoted the ubiquitination of R5/PTG. All these results provided the first functional linkage between malin and TRIM32.

**Figure 7:**
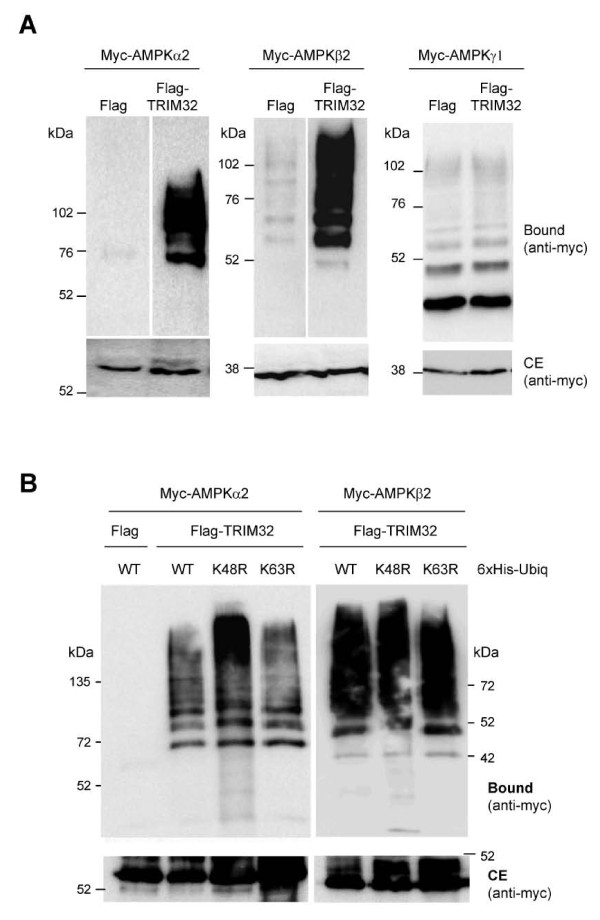
**TRIM32 ubiquitinates another malin substrate such as AMPK subunits**. A) HEK293 cells were co-transfected as in part A of Figure 6 using myc-AMPK subunits α2, β2 or γ1 as a substrate, and either Flag-TRIM32 or empty vector (Flag). Samples were processed and analyzed as in Figure 6. Only in the case of AMPKα2 (left panel) and AMPKβ2 (middle panel), Flag-TRIM32 produced poly-ubiquitinated forms. B) Chain topology of ubiquitinated AMPK subunits. HEK293 cells were co-transfected with myc-AMPKα2 (left panel) or β2 subunit (right panel), 6xHis-tagged ubiquitin constructs (WT, K48R or K63R), and either empty vector (Flag) or Flag-TRIM32. Cell extracts were analyzed as in Figure 6 using anti-myc antibodies.

To determine the extent of this functional link, we decided to study the effect of TRIM32 on another known substrate of the laforin-malin complex, namely the AMP-activated protein kinase (AMPK). AMPK is composed of three subunits (α, β and γ) and we previously demonstrated the specific ubiquitination of all subunits by the laforin-malin complex [16]. Using the same methodology described above, we observed that Flag-TRIM32 produced a robust ubiquitination of both AMPKα and AMPKβ subunits, but not AMPKγ (Figure 7A).

In addition, we focused on the topology of ubiquitin chains produced by TRIM32 in the AMPK subunits. We used different ubiquitin constructs mutated in either Lys48 (K48R) or Lys63 (K63R), unable to oligomerize at those residues. We recently described that the laforin-malin complex promotes the acquisition of K63-linked polyubiquitin chains onto AMPK [16]. Conversely, TRIM32 produced a different polyubiquitin chain topology since it was able to generate ubiquitination in the presence of either K48R- or K63R-ubiquitins (Figure 7B). These results suggest a diversification in the activities of these two E3-ubiquitin ligases, despite sharing common substrates.

**Figure 8:**
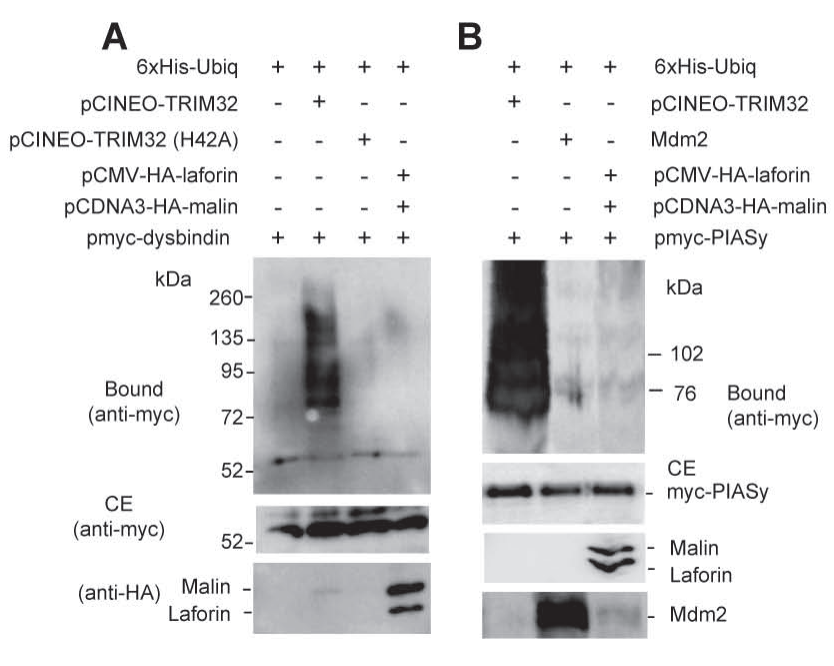
**The laforin-malin complex does not ubiquitinate TRIM32-related substrates such as dysbindin and PIASy**. A) Ubiquitination of dysbindin. HEK293 cells were transfected with 6xHis-tagged ubiquitin construct, pmyc-dysbindin and plasmids encoding TRIM32 [wild type or a catalytically inactive form (H42A)] or plasmids encoding laforin and malin (pCMV-HA-laforin and pCDNA3-HA-malin). Samples were processed and analyzed as in Figure 6. Crude extracts were also analyzed for the presence of laforin and malin using an anti-HA antibody. B) Ubiquitination of PIASy. Similar experiments as those performed in part A) were conducted in cells expressing myc-PIASy. In addition we probed the action of Mdm2, another E3-ubiquitin ligase. Crude extracts were analyzed for the presence of laforin and malin using an anti-HA antibody, and with an anti-Mdm2.

Since TRIM32 can ubiquitinate malin substrates, we decided to investigate whether malin could ubiquitinate TRIM32 substrates. It has been described that TRIM32 ubiquitinates, among other substrates, dysbindin (a protein involved in endosomal-lysosomal trafficking and the genetic aetiology of schizophrenia) [33], and PIASy [Protein Inhibitor of Activated STAT (Signal Transducer and Activator of Transcription) isoform y, an E3-SUMO ligase] [34]. Following a similar approach as described above but using myc-dysbindin and myc-PIASy as substrates, we observed that only wild type TRIM32 but not the laforin-malin complex was able to ubiquitinate myc-dysbindin (Figure 8A) and myc-PIASy (Figure 8B). We also tested another unrelated E3-ubiquitin ligase named Mdm2, an E3 ligase for p53, but we found that it could not ubiquitinate PIASy either, under these conditions (Figure 8B). These results indicate that the functional link between malin and TRIM32 is not completely reciprocal.

## Discussion

Malin is an E3-ubiquitin ligase mutated in Lafora progressive myoclonus epilepsy and interacts with the dual specificity phosphatase laforin. We sought to define the species distribution of malin in order to compare it with the species distribution observed in laforin. We utilized a bioinformatics approach and found that laforin and malin do not correlate in species distribution. Laforin has a broader distribution and is conserved in all vertebrates as well as a select group of protists and invertebrates. Alternatively, malin is only present in vertebrate species and the cephalochordate *B. floridae*, although the ubiquitin system is present in both invertebrates and protists [1]. In addition, these studies revealed that TRIM32, another E3-ubiquitin ligase belonging to the tripartite-motif containing family, is closely related to malin. By means of sequence analyses, we defined the phylogenetic relationship between malin and other members of the TRIM family, and found that TRIM32 is the closest homolog to malin.

Although the evolutionary history of large protein families is complex and difficult to asses, recent studies have established a well defined phylogeny for TRIM proteins [28]. They are divided into Group 1 and Group 2, with Group 2 being more divergent and having evolved more rapidly. Our analyses found that malin is more similar to Group 1 TRIM family members. Comparison of malin with Group 1 members of the TRIM family demonstrate that malin is most similar to TRIM32, and they are more distantly related to TRIM71, TRIM2, and TRIM56. Our results concerning TRIM proteins correlate with the orthology groups of TRIM proteins previously defined by Sardiello et al. [28], although they did not present any data on malin. Furthermore, our analyses demonstrate that TRIM32 and malin appear latter in the evolutionary time-line, since only vertebrate species show orthologs for both proteins. Conversely, TRIM2 and especially TRIM71 are present in species of eukaryotic taxa evolutionarily situated closer to the probable root of the eukaryotic tree. The absence of orthologs in specific vertebrate organisms belonging to the same taxon (for instance, there is a *Takifugu rubripes *ortholog for malin but not a *Danio rerio *one) is probably due to incomplete sequencing of these organisms and not due to gene loss. In addition, the analyses of gene structure and intron-exon boundaries clearly indicate a close relationship between malin and TRIM32.

Despite sequence similarities, malin and TRIM32 have been characterized as E3-ubiquitin ligases involved in different diseases. Both proteins display E3-ubiquitin ligase activity and share the presence of NHL domains at their tertiary structure. The physiological ubiquitination process performed by malin depends strictly on the interaction with laforin, and no oligomerization has been proved to date on malin. On the contrary, TRIM32 requires self-oligomerization in order to perform ubiquitination [35]. Although the conserved domains shared by both proteins show a high degree of identity, the absence of the B-box and coiled-coil domains in malin constitutes an important difference that may reflect different substrate specificities. In order to assess the functional similarities of both proteins, we analysed the capability of TRIM32 to ubiquitinate laforin/malin specific substrates. A first evidence of a functional link between TRIM32 and malin was found in the ubiquitination of R5/PTG: we observed that TRIM32 is able to ubiquitinate this substrate. Next, we tested the action of TRIM32 on AMPK subunits, which are also substrates of the laforin-malin complex. In this case, TRIM32 ubiquitinated only the AMPKα and AMPKβ subunits, whereas malin also ubiquitinates the AMPKγ subunit. These modifications are specific of the action of laforin/malin or TRIM32, since we previously described that another unrelated E3-ubiquitin ligase such as Mdm2 could not ubiquitinated any of these substrates [16]. Analysis of the topology of the ubiquitin chains showed that ubiquitination of AMPKα and AMPKβ by TRIM32 is not dependent on either Lys48 or Lys63 ubiquitin residues, suggesting a different topology to the one produced by malin (K63 ubiquitin-linked chains [16]). Therefore, while there are similarities in substrate preference, there are also differences in both substrate preference and in the types of ubiquitin chains they promote. Further studies are required to fully assess the functional consequences of this novel TRIM32 modification on AMPK activity. We also found that laforin could be ubiquitinated by TRIM32 action (data not shown). However, as we were unable to detect any physical interaction between TRIM32 and any of the malin substrates (R5/PTG, AMPK subunits and laforin), either by yeast two-hybrid analysis or by co-immunoprecipitation methods in mammalian cells, we favour the idea that TRIM32 is a promiscuous E3-ubiquitin ligase that might be using a "hit and run" mechanism to ubiquitinate these substrates. Although our data strongly suggest that TRIM32 can directly ubiquitinate malin substrates, this has not been definitively proved as we did not recapitulate the ubiquitination reaction *in vitro *using purified components; therefore, it might be possible that the activity that we are assigning to TRIM32 could be exerted by a TRIM32-dependent activation of a third E3-ligase.

TRIM32 also ubiquitinates muscular actin [30], dysbindin [33], and the E3-SUMO ligase PIASy [34]. TRIM32 has been related to neuronal differentiation in mice [36] and its deficiency (TRIM32 D487N mutation) is involved in Limb-Girdle muscular dystrophy type 2H (OMIM 254110) [30,35]. It has been postulated that the milder phenotype of patients having this disease may be explained by redundant functions of other E3-ubiquitin ligases performing compensatory TRIM32 functions [34]. Since our structural models of malin and TRIM32 NHL domains show a similar three-dimensional organization, it is possible that malin or other related E3-ubiquitin ligases are capable of compensating the loss of TRIM32 and interact and/or ubiquitinate TRIM32-related substrates. However, no ubiquitination of the TRIM32-specific substrates dysbindin and PIASy by malin was found in our studies. Therefore, further studies will be required to fully assess the capability of malin to compensate TRIM32 loss. Conversely, it has been recently described that malin deficient mice do not present differences in the levels of R5/PTG [15]. Perhaps TRIM32 or another E3-ubiquitin ligase could compensate malin deficiency in terms of ubiquitinating this substrate. In any case, although TRIM32 and malin share some substrates, the pathological outcomes of the deficiency of TRIM32 and malin are very distinct, which indicates that some other factors affect the function of these two E3-ligases in their particular target tissues.

Other mutations in the TRIM32 gene are related to the Bardet-Biedl syndrome type 11 (BBS11, OMIM 209900) [35], whose symptoms include obesity, retinophaty, kidney and heart abnormalities and cognitive deficiency. The mutation responsible for this disease (P130S) is located in the B-box region of the protein. Since this region is absent in malin, comparison of TRIM32 and malin mutants and their abilities to bind putative common interactors like E2-ubiquitin conjugating enzymes would constitute an approach to further asses the functional divergence of both proteins and their linkage to disease. In addition, the study of the E2-conjugating enzymes that coordinate ubiquitin chain elongation in malin- and TRIM32-regulated processes, may lead to a better understanding of the cross-talking of their ubiquitin pathways and its pathological consequences.

## Conclusions

In summary, our results show that malin is an E3-ubiquitin ligase only present in all vertebrate species and a cephalochordate. Malin is related to the TRIM family of proteins, and they likely share a common evolutionary origin. Moreover, malin shares functional features with TRIM32 in that they are both capable of ubiquitinating similar substrates. However, differences at ubiquitin chain topology promoted by each of these proteins point to divergent roles. In addition, malin shows a pattern of species distribution that does not correlate with the species distribution of laforin, which suggest that laforin and malin, in addition to forming a functional complex, may have independent functions.

## Methods

### Bioinformatics analysis

Malin and TRIM orthologs were obtained by performing BLASTP, PHI-BLAST, and PSI-BLAST searches on NCBI non-redundant protein databases. To further refine the searches, they were limited to metazoan and protozoan taxa, and TBLASTN searches in the case of *N. vectensis, B. floridae, C. intestinalis *and *M. brevicollis *(http://www.ncbi.nlm.nih.gov/mapview/). In addition, we analyzed multiple organism-specific databases (additional file 2, Table S1). The BLAST results were analyzed for domain structure and domain boundaries. Structural and domain searches were also performed using SUPERFAMILY [37], PROSITE, and Conserved Domain Database (CDD). Multiple sequence alignments were created using MAFFT [26] and CLUSTALW. Although they gave similar results, we utilized the MAFFT alignments due to recent reviews stating CLUSTALW inferiority [38]. Mid-point rooted maximum likelihood trees were generated from MAFFT generated multiple sequence alignments using PROML from the PHYLIP 3.65 software package and displayed utilizing HYPERTREE [39]. The most appropriated model for these data was assessed using Akaike Information Criterion in ProtTest [40] and the most appropriate model was JTT+G+F. Structural models were created with EsyPred3D [41] and images displayed with PyMOL (http://www.pymol.org). Accession numbers for the protein sequences used in phylogenetic trees and species distribution are included as additional file 7, Table S2.

### Plasmids

Plasmids used in this study were pCMVmyc-AMPKα2, pCMVmyc-AMPKβ2 and pCMVmyc-AMPKγ1 [42]; pCDNA3-HA-malin, pFLAG-laforin, and pCMV-myc-R5/PTG [10]. TRIM32 gene was PCR-amplified from human cDNA using specific primers and PCR, and cloned into pFLAG-CMV6c. When indicated, plasmids pCINEO-TRIM32 and pCINEO-TRIM32 (H42A), expressing respectively wild type and a catalytically inactive form of TRIM32 with a H42A mutation in the RING domain [33] (a generous gift from Dr. Derek J. Blake, Dept. Psychological Medicine, Cardiff University, UK), were also used in the assays. Other plasmids used in this study were: pCMV-mycPIASy (subcloned from plasmid pACT2-PIASy, a gift from Dr. Santiago Rodriguez de Cordoba, Centro de Investigaciones Biológicas, Madrid, Spain); pmyc-dysbindin [33] (from Dr. Derek J. Blake, Dept. Psychological Medicine, Cardiff University, UK), pCMV-6xHisUbiq (from Dr. Manuel Rodriguez, Proteomics Unit, CIC-BioGUNE, Vizcaya, Spain), pCMV-6xHisUbiq K48R and pCMV-6xHisUbiq K63R (from Dr. Christine Blattner, Institute of Toxicology and Genetics, Karlsruhe Institute of Technology, Germany) and pCMV-Mdm2 [9].

### Cell models and culture conditions

Human embryonic kidney (HEK293) cells and mouse embryonic fibroblasts (MEFs) from epm2b-/- mice lacking malin were grown in DMEM (Lonza, Barcelona, Spain) supplemented with 100 units/ml penicillin, 100 μg/ml streptomycin, 2 mM glutamine, 10% inactivated fetal bovine serum (Invitrogen, Madrid, Spain). 1.5 × 10^6 ^cells were plated onto 60 mm-diameter culture dishes the day before transfection. Cells were transfected with 1 μg of each plasmid using Lipofectamine 2000 (Invitrogen, Madrid, Spain).

### Analysis of *in vivo *ubiquitination

To study ubiquitination in intact cells, HEK293 cells were transfected with plasmids pCMV-6xHisUbiq (encoding a modified ubiquitin, tagged with 6xHis residues), pCMVmyc plasmids encoding the protein of interest and plasmids encoding different E3-ubiquitin ligases, using the Lipofectamine 2000 reagent (Invitrogen, Madrid, Spain), according to the manufacturer's instructions. After 36 hours of transfection, cells were lysed in buffer A (6 M guanidinium-HCl, 0.1 M sodium phosphate, 0.1 M Tris-HCl, pH 8.0). Four mg of protein of a clarified extract (CE; 12,000 × g, 15 min) were incubated in 100 μl TALON column (Clontech, Barcelona, Spain) in the presence of 10 mM imidazole, for 3 hours at room temperature on a rocking platform, to purify His-tagged proteins. The column was then successively washed with 2 ml each of buffer B (buffer A plus 10 mM imidazole), buffer C (buffer B, but with 8 M urea instead of 6 M guanidinium-HCl) and four more times with buffer C adjusted to pH 6.0. Bound proteins (Bound) were eluted with 50 μl of 2× Laemmli's sample buffer and analyzed by Western blotting using appropriated antibodies. When indicated, plasmids pCMV-6xHisUbiq K48R and pCMV-6xHisUbiq K63R were used in the assay instead of plasmid pCMV-6xHisUbiq.

### Immunodetection

HEK293 and epm2b-/- MEF cells were transfected with the corresponding plasmids. Twenty-four hours after transfection, cells were scraped on ice in lysis buffer [10 mM TrisHCl pH 8; 150 mM NaCl, 15 mM EDTA; 0.6 M sucrose, 0.5% nonidet P-40 (NP-40), complete protease inhibitor cocktail (Roche Biotech, Barcelona, Spain), 1 mM PMSF, 50 mM NaF and 5 mM Na_2_P_2_O_7_]. Cells were lysed by repeated passage through a 25-gauge needle. Twenty-five μg of total protein from the soluble fraction of cell lysates were analyzed by SDS-PAGE and western blotting using appropriate antibodies: anti-myc, anti-FLAG and anti-HA (Sigma, Madrid, Spain), and anti-Mdm2 (Santa Cruz Biotechnology, Madrid, Spain).

## List of Abbreviations

AMPK: AMP-activated protein kinase; NHL: a protein domain present in Ncl-1: HT2A and Lin-41 proteins; PIASy: protein inhibitor of activated STAT (signal transducer and activator of transcription); R5/PTG: protein targeting to glycogen; RING: really interesting new gene; TRIM: Tripartite motif-containing protein.

## Authors' contributions

CR-M carried out the molecular genetic studies, participated in the sequence alignment and drafted the manuscript; D.M. performed the ubiquitination experiments of AMPK using TRIM32; SV. and TR. performed the ubiquitination of PIASy using TRIM32; TMB. carried out molecular genetic studies and participated in the sequence alignment; MSG. and PS. participated in the design of the study, analyzed the data and helped to draft the manuscript. All authors read and approved the final manuscript.

## Supplementary Material

Additional file 1 **Alignment of malin orthologs**. Protein sequences from all vertebrate malin orthologs were utilized to generate an alignment in MAFFT. This alignment was utilized for further analyses. Click here for file

Additional file 2 **Genomes investigated for presence of malin**. Click here for file

Additional file 3 **Proteins from Archea genomes containing a RING domain**. Putative proteins with a RING domain in eukaryotic, bacterial, and archea genomes were identified using the SUPERFAMILY database. Hundreds of proteins in eukaryotic and bacterial genomes were identified and were not further analyzed. Three positive archea hits were subsequently analyzed using PFAM, InterProScan, and SMART for confirmation. In addition, predicted secondary structure was analyzed using JPRED and PSIPRED. Predicted consensus secondary structure is displayed over the sequences with arrows representing β-sheets and ovals representing α-helices. The conserved cysteine residues are highlighted in red. Protein identification numbers are: *Nitrosopumilus maritimus*-YP_001581408.1, *Methanosarcina acetivorans*-NP_617583.1, and *Methanosarcina barkeri*-YP_306007.1. Click here for file

Additional file 4 **Proteins from Archea genomes containing NHL domains**. Description: Putative proteins with NHL domains in eukaryotic, bacterial, and archea genomes were identified using the SUPERFAMILY database. Hundreds of proteins in eukaryotic and bacterial genomes were identified and were not further analyzed. Three positive archea hits were subsequently analyzed using PFAM, InterProScan, and SMART for confirmation. In addition, predicted secondary structure was analyzed using JPRED and PSIPRED. Predicted consensus secondary structure is displayed over the sequences with arrows representing β-sheets. The predicted NHL domains are highlighted with green boxes. Protein identification numbers are: *Halalkallcoccus*-YP_685176.1, *Halomicrobium*-YP_003178756.1, and archaeon RC-I-YP_003736383.1. Click here for file

Additional file 5 **Alignment of malin and TRIM orthologs**. Protein sequences of malin and TRIM sequences were utilized to generate an alignment in MAFFT. This alignment was utilized for further analyses. Click here for file

Additional file 6 **Known and predicted intron-exon boundaries for malin, TRIM2, TRIM32, TRIM56, and TRIM71 genes were determined using the UC-Santa Cruz genome browser**. A vertebrate class or genus species name is given for each gene. Each grey box represents a single exon. The length of each exon is depicted to scale. Click here for file

Additional file 7 **Accession numbers for the sequences used for the generation of **Figures 2, 3 and 4. Click here for file
