# Supplementary figures and images for: Lafora disease E3-ubiquitin ligase malin is related to TRIM32 at both the phylogenetic and functional level
Source: BMC Evol Biol. 2011 Jul 28;11:225. doi: 10.1186/1471-2148-11-225 (PMC3160408; doi:10.1186/1471-2148-11-225)

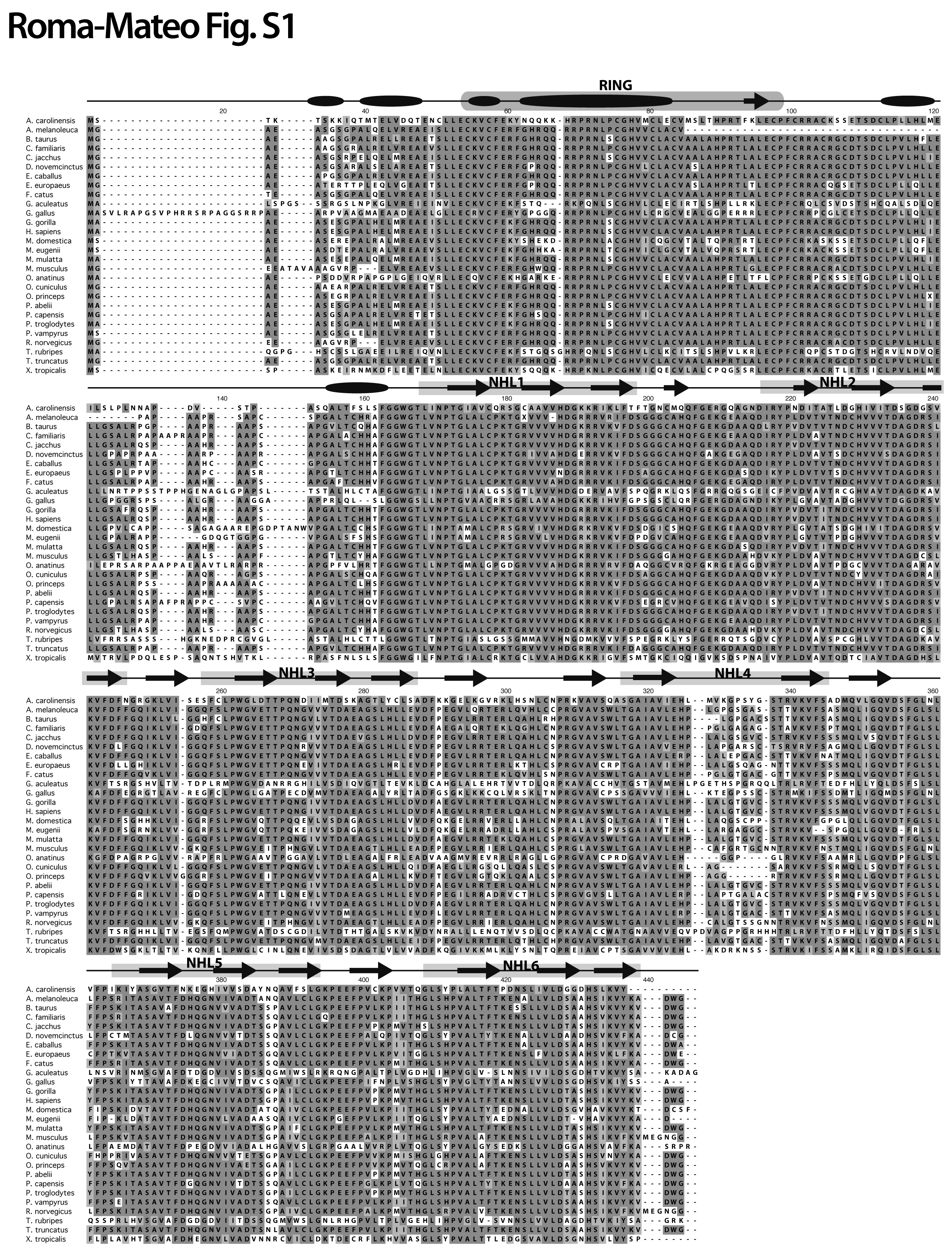

Supplement: Additional file 1 — Alignment of malin orthologs. Protein sequences from all vertebrate malin orthologs were utilized to generate an alignment in MAFFT. This alignment was utilized for further analyses. [file 1471-2148-11-225-S1.TIFF]

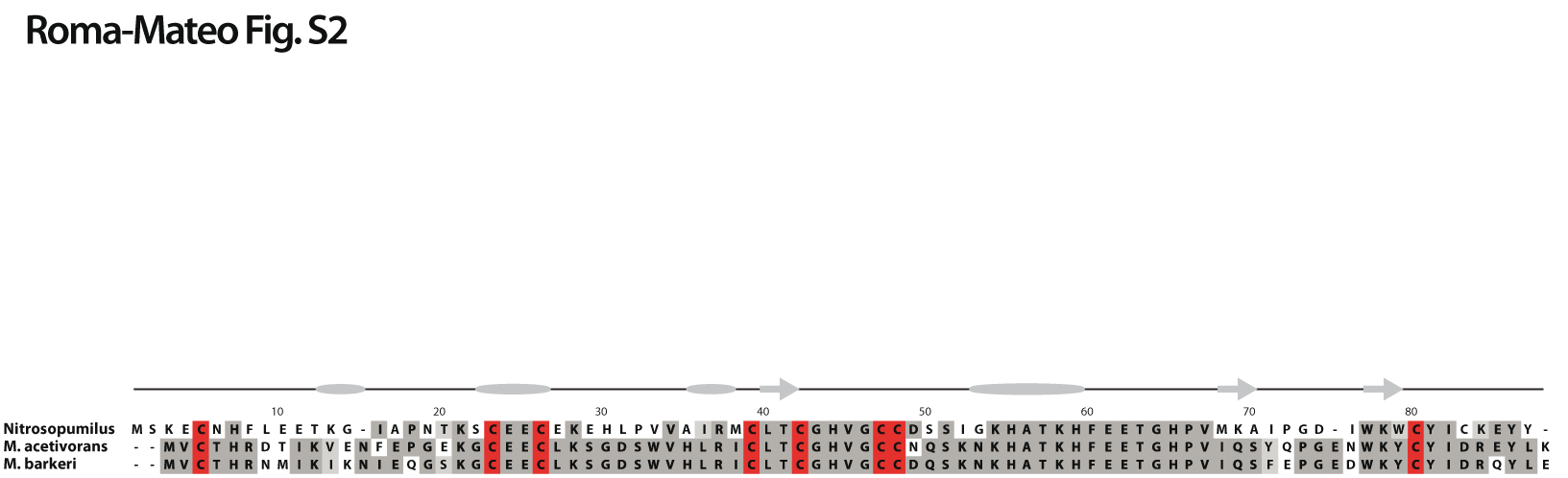

Supplement: Additional file 3 — Proteins from Archea genomes containing a RING domain. Putative proteins with a RING domain in eukaryotic, bacterial, and archea genomes were identified using the SUPERFAMILY database. Hundreds of proteins in eukaryotic and bacterial genomes were identified and were not further analyzed. Three positive archea hits were subsequently analyzed using PFAM, InterProScan, and SMART for confirmation. In addition, predicted secondary structure was analyzed using JPRED and PSIPRED. Predicted consensus secondary structure is displayed over the sequences with arrows representing β-sheets and ovals representing α-helices. The conserved cysteine residues are highlighted in red. Protein identification numbers are: Nitrosopumilus maritimus-YP_001581408.1, Methanosarcina acetivorans-NP_617583.1, and Methanosarcina barkeri-YP_306007.1. [file 1471-2148-11-225-S3.TIFF]

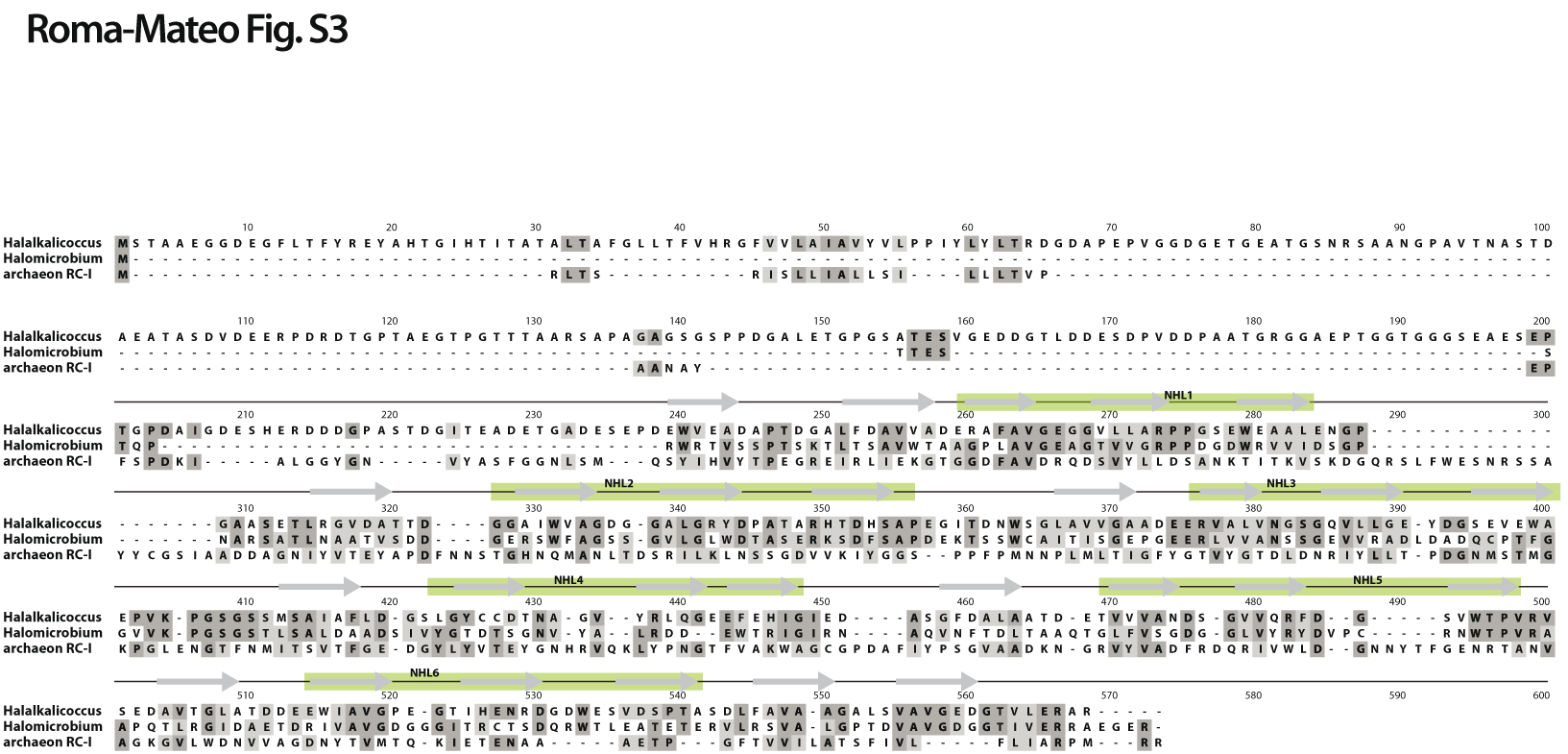

Supplement: Additional file 4 — Proteins from Archea genomes containing NHL domains. Description: Putative proteins with NHL domains in eukaryotic, bacterial, and archea genomes were identified using the SUPERFAMILY database. Hundreds of proteins in eukaryotic and bacterial genomes were identified and were not further analyzed. Three positive archea hits were subsequently analyzed using PFAM, InterProScan, and SMART for confirmation. In addition, predicted secondary structure was analyzed using JPRED and PSIPRED. Predicted consensus secondary structure is displayed over the sequences with arrows representing β-sheets. The predicted NHL domains are highlighted with green boxes. Protein identification numbers are: Halalkallcoccus-YP_685176.1, Halomicrobium-YP_003178756.1, and archaeon RC-I-YP_003736383.1. [file 1471-2148-11-225-S4.TIFF]

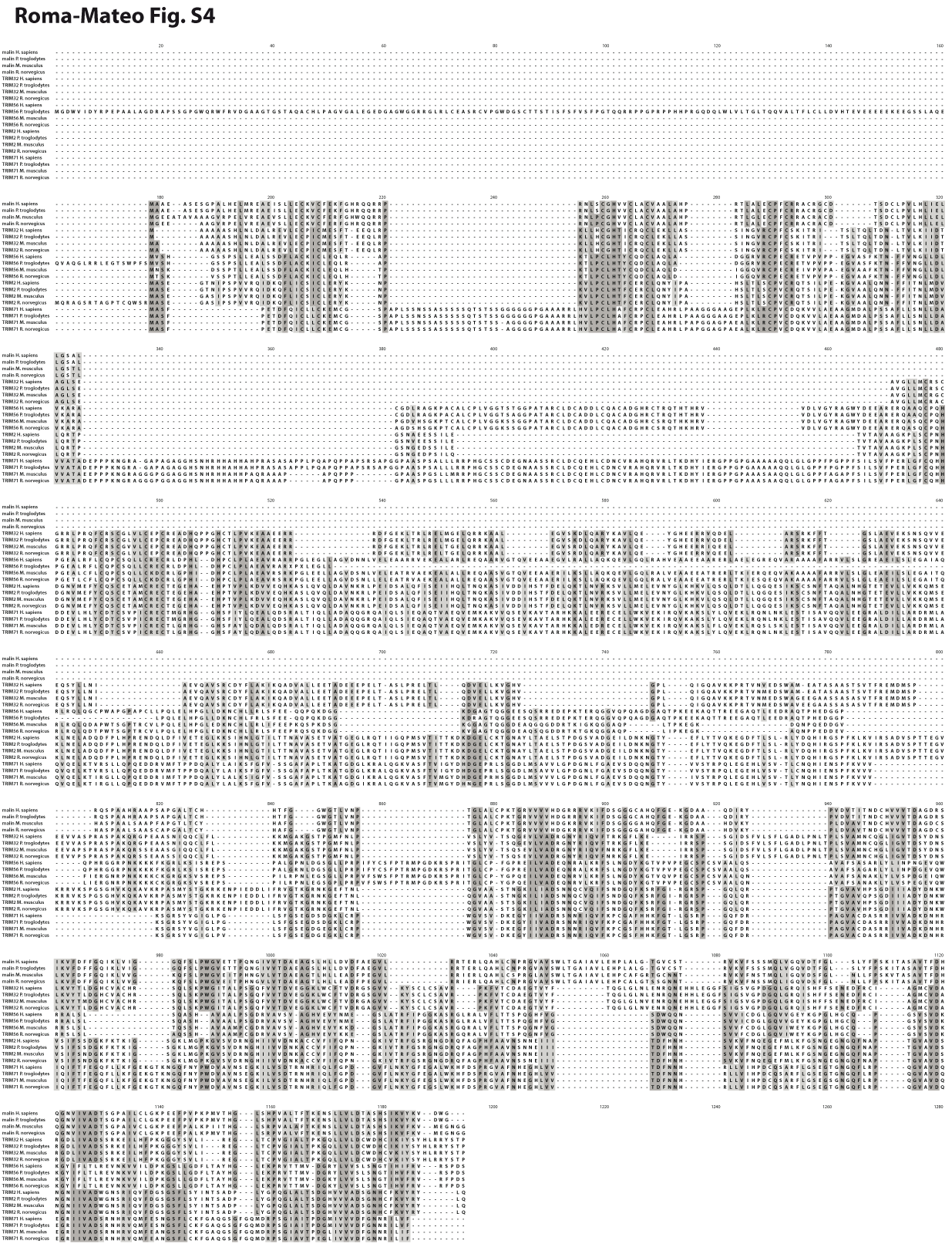

Supplement: Additional file 5 — Alignment of malin and TRIM orthologs. Protein sequences of malin and TRIM sequences were utilized to generate an alignment in MAFFT. This alignment was utilized for further analyses. [file 1471-2148-11-225-S5.TIFF]

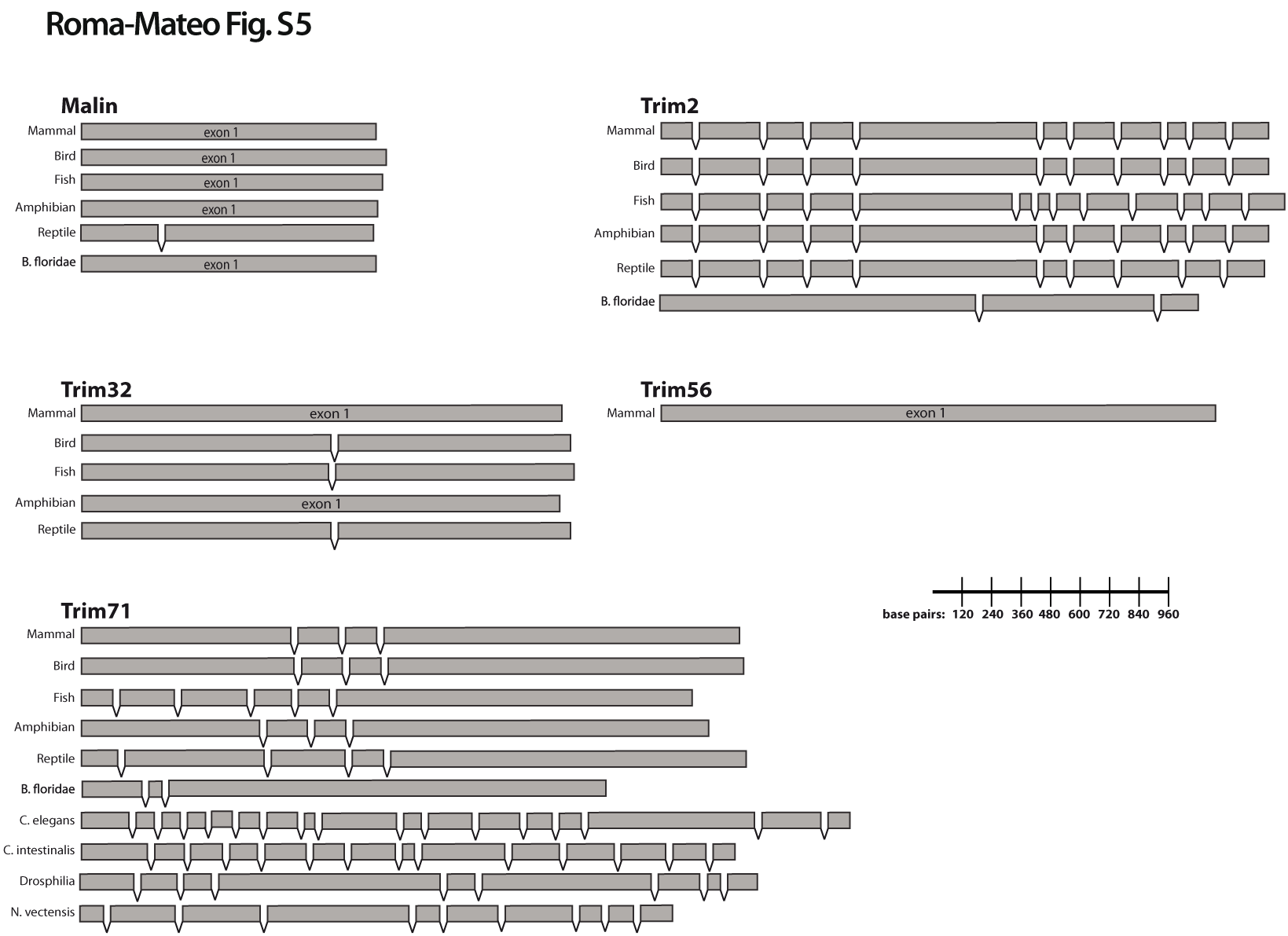

Supplement: Additional file 6 — Known and predicted intron-exon boundaries for malin, TRIM2, TRIM32, TRIM56, and TRIM71 genes were determined using the UC-Santa Cruz genome browser. A vertebrate class or genus species name is given for each gene. Each grey box represents a single exon. The length of each exon is depicted to scale. [file 1471-2148-11-225-S6.TIFF]
