# Supplementary material for: Lafora disease E3-ubiquitin ligase malin is related to TRIM32 at both the phylogenetic and functional level
Source: BMC Evol Biol. 2011 Jul 28;11:225. doi: 10.1186/1471-2148-11-225 (PMC3160408; doi:10.1186/1471-2148-11-225)
Supplement: Additional file 2 — Genomes investigated for presence of malin. [file 1471-2148-11-225-S2.DOC]

**Supplementary Table SI:** Genomes investigated for presence of malin.

**I. Eukaryotes**

**A. Amoebozoa**

**1. Tubulinea**

**2. Flabellinea**

**3. Stereomyxida**

**4. Acanthamoebidae**

**5. Entamoebida**

*Entamoeba dispar* lacks malin

*Entamoeba histolytica* lacks malin

*Entamoeba invadens* lacks malin

*Entamoeba moshkovskii* lacks malin

*Entamoeba terrapinae* lacks malin

**6. Mastigamoebidae**

**7. *Pelomyxa***

**8. Eumycetozoa**

*Dictyostelium discoideum* lacks malin

**B. Opisthokonta**

**1. Fungi**

*Ajellomyces capsulatus* lacks malin

*Ascosphaera apis* lacks malin

*Ashbya gossypii* lacks malin

*Aspergillus clavatus* lacks malin

*Aspergillus flavus*  lacks malin

*Aspergillus fumigatus* lacks malin

*Aspergillus nidulans* lacks malin

*Aspergillus terreus* lacks malin

*Batrachochytrium dendrobatidis* lacks malin

*Botryotinia fuckelinana* lacks malin

*Candida albicans* lacks malin

*Candida glbrata* lacks malin

*Candida tropicalis* lacks malin

*Chaetomium globosum* lacks malin

*Clavispora lusitaniae* lacks malin

*Coccidioides immitis* lacks malin

*Coccidioides posadasii* lacks malin

*Coprinopsis cinerea okayama* lacks malin

*Cryptococcus neoformans* sp. lacks malin

*Debaryomyces hansenii* lacks malin

*Encephalitozoon cuniculi* lacks malin

*Eremothecium gossypii* lacks malin

*Fusarium oxysporum* lacks malin

*Gibberella monoiliformis* lacks malin

*Gibberella zeae* lacks malin

*Kluyveromyces lactis* lacks malin

*Kluyveromyces waltii* lacks malin

*Laccaria bicolor* lacks malin

*Lodderomyces elongisporus* lacks malin

*Magnaporthe grisea* lacks malin

*Malassezia furfur* lacks malin

*Mycosphaerella graminocola* lacks malin

*Neosartorya fischeri* lacks malin

*Nectria haematococca* lacks malin

*Neurospora crassa* lacks malin

*Phaeosphaeria nodorum* lacks malin

*Phanerochaete chrysosporium* lacks malin

*Pichia guilliermondii* lacks malin

*Pichia stipitis* lacks malin

*Penicillium marneffei* lacks malin

*Pneumocystis carnii* lacks malin

*Podospora anserina* lacks malin

*Puccinia graminis* lacks malin

*Pyrenophora tritici-repentis* lacks malin

*Rhizopus oryzae* lacks malin

*Saccharomyces bayanus* lacks malin

*Saccharomyces castellii* lacks malin

*Saccharomyces cerevisiae* lacks malin

*Saccharomyces kluyveri* lacks malin

*Saccharomyces kudriavzevii* lacks malin

*Saccharomyces mikatae* lacks malin

*Saccharomyces paradoxus* lacks malin

*Schizosaccharomyces japonicus* lacks malin

*Schizosaccharomyces pombe* lacks malin

*Sclerotinia sclerotiorum* lacks malin

*Sporobolomyces roseus* lacks malin

*Talaromyces stipitatus* lacks malin

*Trichoderma atroviride* lacks malin

*Trichoderma reesei* lacks malin

*Trichoderma virens* lacks malin

*Uncinocarpus reesii* lacks malin

*Ustilago maydis* lacks malin

*Vanderwaltozyma polyspora* lacks malin

*Yarrowia lipolytica* lacks malin

**2. Mesomycetozoa**

**3. Choanomonada**

*Monosiga brevicollis* lacks malin

**4. Metazoa**

*Capitella sp.* lacks malin

*Helobdella robusta* lacks malin

*Aedes aegypti* lacks malin

*Anopheles gambiae* lacks malin

*Apis mellifera* lacks malin

*Bombyx mori* lacks malin

*Culex pipens* lacks malin

*Daphnia pulex* lacks malin

*Drosophila ananassae* lacks malin

*Drosophila erecta* lacks malin

*Drosophila grimshawi* lacks malin

*Drosophila mojavensis* lacks malin

*Drosophila persimilis* lacks malin

*Drosophila pseudoobscura* lacks malin

*Drosophila sechellia* lacks malin

*Drosophila simulans* lacks malin

*Drosophila virilis* lacks malin

*Drosophila willistoni* lacks malin

*Drosophila yakuba* lacks malin

*Glossina morsitans* lacks malin

*Nasonia vitripennis* lacks malin

*Pediculus humanus corporis* lacks malin

*Tribolium castaneum* lacks malin

*Branchiostoma floridea*  **has malin**

*Hydra sp.* lacks malin

*Nematostella vectensis* lacks malin

*Lottia gigantea* lacks malin

*Ascaris lumbicoides* lacks malin

*Brugia malayi* lacks malin

*Caenorhabditis briggsae* lacks malin

*Caenorhabditis brenneri* lacks malin

*Caenorhabditis elegans* lacks malin

*Caenorhabditis remanei* lacks malin

*Haemonchus contortus* lacks malin

*Trichoplax sp.* lacks malin

*Fasciola hepatica* lacks malin

*Schistosoma mansoni* lacks malin

*Aplysia californica* lacks malin

*Ciona intestinalis* lacks malin

*Ciona savignyi* lacks malin

*Strongylocentrotus purpuratus* lacks malin

**vertebrates**

*Anolis carolinensis* mammal, has malin

*Ailuropoda melanoleuca* mammal, has malin

*Bos taurus* mammal, has malin

*Canis familiaris* mammal, has malin

*Callithrix jacchus* mammal, has malin

*Cavia porcellus* mammal, incomplete genome, has malin

*Dasypus novemcinctus* mammal, has malin

*Echinops telfairi* mammal, incomplete genome, has malin

*Equus caballus* mammal, has malin

*Erinaceus eruopaeus* mammal, has malin

*Felis catus* mammal, has malin

*Gallus gallus* aves, has malin

*Gasterosteus aculeatus* osteichthyes, has malin

*Gorilla gorilla* mammal, has malin

*Homo sapiens* mammal, has malin

*Loxodonta africana* mammal, incomplete genome, has malin

*Macaca mulatta* mammal, has malin

*Macropus eugenii* mammal, has malin

*Microcebus murinus* mammal, incomplete genome, has malin

*Monodelphis domestica* mammal, has malin

*Mus musculus* mammal, has malin

*Myotis lucifugus* mammal, incomplete genome, has malin

*Ochotona princeps* osteichthyes, has malin

*Ornithorhynchus anatinus* mammal, has malin

*Oryctolagus cuniculus* mammal, incomplete genome, has malin

*Otolemur garnettii* mammal, incomplete genome, has malin

*Pan troglodytes* mammal, has malin

*Pongo abelii* mammal, has malin

*Procavia capensis* mammal, has malin

*Pteropus vampyrus* mammal, has malin

*Rattus norvegicus* mammal, has malin

*Sorex araneus* mammal, incomplete genome, has malin

*Sus scrofa* mammal, incomplete genome, has malin

*Takifugu rubripes* osteichthyes, has malin

*Tursiops truncatus* mammal, has malin

*Xenopus tropicalis* mammal, has malin

**C. Rhizaria**

**1. Cercozoa**

*Bigelowiella natans* lacks malin

*Phytophthora infestans* lacks malin

*Phytophthora ramorum* lacks malin

*Phytophthora sojae* lacks malin

**2. Haplosporidia**

**3. Foraminifera**

**4. *Gromia***

**5. Radiolaria**

**D. Archaeplastida**

**1. Glaucophyta**

*Cyanophora paradoxa* lacks malin

**2. Rhodophyceae**

*Cyanidioschyzon merolae* lacks malin

*Galdieria sulphuraria* lacks malin

**3. Chloroplastida**

*Arabidopsis thaliana* lacks malin

*Aquilegia sp.* lacks malin

*Chlamydomonas reinhardtii* lacks malin

*Citrus sinensis* lacks malin

*Medicago truncatula* lacks malin

*Micromonas sp.* lacks malin

*Oryza sativa* lacks malin

*Osterococcus lucimarinus* lacks malin

*Ostreococcus tauri* lacks malin

*Phaseolus vulgaris* lacks malin

*Physcomitrella patens* lacks malin

*Selaginella moellendorffii* lacks malin

*Solanum lycopersicum* lacks malin

*Solanum tuberosum* lacks malin

*Sorghum bicolor* lacks malin

*Triticum aestivum*  lacks malin

*Vitis vinifera* lacks malin

*Volvox carteri* lacks malin

*Zea mays* lacks malin

**E. Chromalveolata**

**1.Cryptophyceae**

*Guillardia theta* lacks malin

*Hemiselmis andersenii* lacks malin

**2. Haptophyta**

*Emiliania huxleyi* lacks malin

**3. Stramenopiles**

*Hyaloperonospora parasitica* lacks malin

*Thalassiosira pseudonana* lacks malin

*Phaeodactylum tricornutum* lacks malin

**4. Alveolata**

*Babesia bovis* lacks malin

*Babesia bigemina* lacks malin

*Babesia malayi* lacks malin

*Cryptosporidium parvum* lacks malin

*Cryptosporidium hominis* lacks malin

*Eimeria tenella* lacks malin

*Encephalitozoon cuniculi* lacks malin

*Neospora caninum* lacks malin

*Paramecium tetraurelia* lacks malin

*Perkinsus marinus* lacks malin

*Plasmodium berghei* lacks malin

*Plasmodium chabaudi* lacks malin

*Plasmodium falciparum* lacks malin

*Plasmodium gallinaceum* lacks malin

*Plasmodium knowlesi* lacks malin

*Plasmodium reichenowi* lacks malin

*Plasmodium vivax* lacks malin

*Plasmodium yhoelii yoelii* lacks malin

*Sarcocystis neurona* lacks malin

*Theileria annulata* lacks malin

*Theileria parva* lacks malin

*Tetrahymena thermophila* lacks malin

*Toxoplasma gondii* lacks malin

**F. Excavata**

**1. Fornicata**

*Giardia lamblia* lacks malin

**2. *Malawimonas***

**3. Parabasalia**

*Trichomonas vaginalis* lacks malin

**4. Preaxostyla**

**5. Jakobida**

**6. Heterolobosea**

*Naegleria gruberi* lacks malin

**7. Euglenozoa**

*Crithidia deanei*  lacks malin

*Leishmania braziliensis* lacks malin

*Leishmania infantum* lacks malin

*Leishmania major* lacks malin

*Leptomonas seymouri* lacks malin

*Trypanosoma brucei* lacks malin

*Trypanosoma congolense* lacks malin

*Trypanosoma cruzi* lacks malin

*Trypanosoma gambiense* lacks malin

**II. Prokaryotes--Archaea and Eubacteria**

All 1408 microbial genomes in NCBI also lack malin
