# Supplementary material for: Lafora disease E3-ubiquitin ligase malin is related to TRIM32 at both the phylogenetic and functional level
Source: BMC Evol Biol. 2011 Jul 28;11:225. doi: 10.1186/1471-2148-11-225 (PMC3160408; doi:10.1186/1471-2148-11-225)
Supplement: Additional file 7 — Accession numbers for the sequences used for the generation of Figures 2, 3 and 4. [file 1471-2148-11-225-S7.DOC]

**Supplementary Table SII:** Accession numbers for the protein sequences used in phylogenetic trees and species distribution.

| **ORGANISM** | **PROTEIN** | **ACCESION NUMBER** |
| --- | --- | --- |
|  |  |  |
| *Homo sapiens* | MALIN | [NP_940988.2](http://www.ncbi.nlm.nih.gov/protein/40255283?report=genbank&log$=prottop&blast_rank=3&RID=778B59ZR01R) |
| *Homo sapiens* | TRIM32 | [NP_001093149.1](http://www.ncbi.nlm.nih.gov/protein/153791514?report=genbank&log$=prottop&blast_rank=2&RID=77988EZM01S) |
| *Homo sapiens* | TRIM71 | [NP_001034200.1](http://www.ncbi.nlm.nih.gov/protein/84993742?report=genbank&log$=prottop&blast_rank=1&RID=776EADV301S) |
| *Homo sapiens* | TRIM56 | [NP_112223.1](http://www.ncbi.nlm.nih.gov/protein/30794216?report=genbank&log$=prottop&blast_rank=3&RID=778M5WRV01R) |
| *Homo sapiens* | TRIM2 | [NP_001123539.1](http://www.ncbi.nlm.nih.gov/protein/194248081?report=genbank&log$=prottop&blast_rank=3&RID=773PM3N901R) |
| *Homo sapiens* | LAFORIN | [NP_005661.1](http://www.ncbi.nlm.nih.gov/protein/11321613?report=genbank&log$=prottop&blast_rank=7&RID=779YRPJ6013) |
|  |  |  |
| *Mus musculus* | MALIN | NP_780549.1 |
| *Mus musculus* | TRIM32 | [EDL31081.1](http://www.ncbi.nlm.nih.gov/protein/148699134?report=genbank&log$=prottop&blast_rank=1&RID=779MX7DM016) |
| *Mus musculus* | TRIM71 | [NP_001035968.1](http://www.ncbi.nlm.nih.gov/protein/109948300?report=genbank&log$=prottop&blast_rank=2&RID=774CP70M01R) |
| *Mus musculus* | TRIM56 | [NP_958761.1](http://www.ncbi.nlm.nih.gov/protein/41235779?report=genbank&log$=prottop&blast_rank=13&RID=778M5WRV01R) |
| *Mus musculus* | TRIM2 | [NP_109631.1](http://www.ncbi.nlm.nih.gov/protein/14010849?report=genbank&log$=prottop&blast_rank=1&RID=773WP08W016) |
| *Mus musculus* | LAFORIN | [NP_034276.2](http://www.ncbi.nlm.nih.gov/protein/116063575?report=genbank&log$=prottop&blast_rank=2&RID=779YRPJ6013) |
|  |  |  |
| *Pan troglodytes* | MALIN | [XP_001170828.1](http://www.ncbi.nlm.nih.gov/protein/114605640?report=genbank&log$=prottop&blast_rank=1&RID=778HW8CN01R) |
| *Pan troglodytes* | TRIM32 | [XP_001156898.1](http://www.ncbi.nlm.nih.gov/protein/114626363?report=genbank&log$=prottop&blast_rank=3&RID=77988EZM01S) |
| *Pan troglodytes* | TRIM71 | [XP_516352.2](http://www.ncbi.nlm.nih.gov/protein/114585883?report=genbank&log$=prottop&blast_rank=1&RID=776P538A01S) |
| *Pan troglodytes* | TRIM56 | [XP_527840.2](http://www.ncbi.nlm.nih.gov/protein/114615086?report=genbank&log$=prottop&blast_rank=15&RID=778M5WRV01R) |
| *Pan troglodytes* | TRIM2 | [XP_001154681.1](http://www.ncbi.nlm.nih.gov/protein/114596401?report=genbank&log$=prottop&blast_rank=1&RID=773PM3N901R) |
| *Pan troglodytes* | LAFORIN | [XP_001161292.1](http://www.ncbi.nlm.nih.gov/protein/114609754?report=genbank&log$=prottop&blast_rank=1&RID=77B2KCPN011) |
|  |  |  |
| *Macaca mulatta* | TRIM56 | [XP_001107405.1](http://www.ncbi.nlm.nih.gov/protein/109066171?report=genbank&log$=prottop&blast_rank=1&RID=77T89KCX01P) |
| *Macaca mulatta* | MALIN | XP_001097330 |
| *Macaca mulatta* | TRIM2 | XP_001086769 |
| *Macaca mulatta* | TRIM32 | XP_001098591 |
| *Macaca mulatta* | TRIM71 | XP_001098706 |
|  |  |  |
| *Rattus norvegicus* | MALIN | [NP_954706.1](http://www.ncbi.nlm.nih.gov/protein/40385887?report=genbank&log$=prottop&blast_rank=1&RID=77TMBU70011) |
| *Rattus norvegicus* | TRIM32 | [NP_001012103.1](http://www.ncbi.nlm.nih.gov/protein/58865776?report=genbank&log$=prottop&blast_rank=10&RID=77THAHTT01P) |
| *Rattus norvegicus* | TRIM71 | [NP_001178730.1](http://www.ncbi.nlm.nih.gov/protein/300796713?report=genbank&log$=prottop&blast_rank=5&RID=77THAHTT01P) |
| *Rattus norvegicus* | TRIM56 | [XP_001076813.2](http://www.ncbi.nlm.nih.gov/protein/293341115?report=genbank&log$=prottop&blast_rank=1&RID=77TA9HM701P) |
| *Rattus norvegicus* | TRIM2 | [NP_001102022.1](http://www.ncbi.nlm.nih.gov/protein/157818847?report=genbank&log$=prottop&blast_rank=1&RID=77TCJRTN015) |
|  |  |  |
| *A. melanoleuca* | TRIM56 | [XP_002930649.1](http://www.ncbi.nlm.nih.gov/protein/301791351?report=genbank&log$=prottop&blast_rank=1&RID=77SDVN9M014) |
| *A. melanoleuca* | TRIM2 | [XP_002913903.1](http://www.ncbi.nlm.nih.gov/protein/301756096?report=genbank&log$=prottop&blast_rank=2&RID=77SPCAXD015) |
| *A. melanoleuca* | MALIN | XP_002922001 |
| *A. melanoleuca* | TRIM32 | EFB19283 |
| *A. melanoleuca* | TRIM71 | EFB27835 |
|  |  |  |
| *C. familiaris* | TRIM56 | [XP_536857.2](http://www.ncbi.nlm.nih.gov/protein/73957817?report=genbank&log$=prottop&blast_rank=1&RID=77T3XDM701R) |
| *C. familiaris* | MALIN | NP_001006650 |
| *C. familiaris* | TRIM32 | XP_853704 |
|  |  |  |
| *B. taurus* | TRIM56 | [XP_875454.2](http://www.ncbi.nlm.nih.gov/protein/119917130?report=genbank&log$=prottop&blast_rank=1&RID=77SHP1AS015) |
| *B. taurus* | TRIM2 | [NP_001077204.1](http://www.ncbi.nlm.nih.gov/protein/139948811?report=genbank&log$=prottop&blast_rank=1&RID=77SVDUA001P) |
| *B. Taurus* | MALIN | XP_582943 |
| *B. Taurus* | TRIM32 | NP_001069292 |
| *B. taurus* | TRIM71 | XP_002707823 |
|  |  |  |
| *E. caballus* | TRIM56 | [XP_001492544.1](http://www.ncbi.nlm.nih.gov/protein/149756018?report=genbank&log$=prottop&blast_rank=1&RID=77SFX37401P) |
| *E. caballus* | TRIM2 | [XP_001501223.1](http://www.ncbi.nlm.nih.gov/protein/149698139?report=genbank&log$=prottop&blast_rank=1&RID=77ST75EE01R) |
| *E. caballus* | MALIN | XP_001496609 |
| *E. caballus* | TRIM32 | XP_001504946 |
| *E. caballus* | TRIM71 | XP_001916815 |
|  |  |  |
| *T. nigroviridis* | TRIM2 | [CAF96577.1](http://www.ncbi.nlm.nih.gov/protein/47216281?report=genbank&log$=prottop&blast_rank=1&RID=77T02F7701R) |
| *T. nigroviridis* | TRIM32 | CAG12414 |
|  |  |  |
| *T. guttata* | TRIM2 | [XP_002198438.1](http://www.ncbi.nlm.nih.gov/protein/224049634?report=genbank&log$=prottop&blast_rank=1&RID=77SY01WK011) |
| *T. guttata* | TRIM71 | XP_002196047 |
|  |  |  |
| *Monodelphis domestica* | MALIN | [XP_001376254.1](http://www.ncbi.nlm.nih.gov/protein/126322237?report=genbank&log$=prottop&blast_rank=1&RID=778B59ZR01R) |
| *Monodelphis domestica* | TRIM32 | [XP_001364618.1](http://www.ncbi.nlm.nih.gov/protein/126293989?report=genbank&log$=prottop&blast_rank=1&RID=779K2E0W013) |
| *Monodelphis domestica* | TRIM2 | [XP_001363305.1](http://www.ncbi.nlm.nih.gov/protein/126330157?report=genbank&log$=prottop&blast_rank=1&RID=774499GH016) |
| *Monodelphis domestica* | LAFORIN | [XP_001381051.1](http://www.ncbi.nlm.nih.gov/protein/126311178?report=genbank&log$=prottop&blast_rank=4&RID=77A7PEZJ01R) |
|  |  |  |
| *Gallus gallus* | MALIN | [XP_426034.2](http://www.ncbi.nlm.nih.gov/protein/118086373?report=genbank&log$=prottop&blast_rank=1&RID=778EJMHJ011) |
| *Gallus gallus* | TRIM71 | [CAJ32595.1](http://www.ncbi.nlm.nih.gov/protein/76665726?report=genbank&log$=prottop&blast_rank=1&RID=776WGFU201S) |
| *Gallus gallus* | TRIM2 | [XP_420365.2](http://www.ncbi.nlm.nih.gov/protein/118089745?report=genbank&log$=prottop&blast_rank=1&RID=773ZPB0D01R) |
| *Gallus gallus* | LAFORIN | [NP_001026240.1](http://www.ncbi.nlm.nih.gov/protein/71894761?report=genbank&log$=prottop&blast_rank=19&RID=77AER714013) |
|  |  |  |
| *Xenopus laevis* | TRIM71 | [XP_002937892.1](http://www.ncbi.nlm.nih.gov/protein/301616912?report=genbank&log$=prottop&blast_rank=1&RID=777H9BWJ013) |
| *Xenopus laevis* | TRIM2 | [NP_001086096.1](http://www.ncbi.nlm.nih.gov/protein/148227830?report=genbank&log$=prottop&blast_rank=5&RID=7748F29C01S) |
| *Xenopus laevis* | LAFORIN | [NP_001085689.1](http://www.ncbi.nlm.nih.gov/protein/148228643?report=genbank&log$=prottop&blast_rank=3&RID=77A7PEZJ01R) |
|  |  |  |
| *Danio rerio* | TRIM32 | [NP_001107066.1](http://www.ncbi.nlm.nih.gov/protein/165972387?report=genbank&log$=prottop&blast_rank=1&RID=77988EZM01S) |
| *Danio rerio* | TRIM71 | [XP_690252.4](http://www.ncbi.nlm.nih.gov/protein/292621983?report=genbank&log$=prottop&blast_rank=4&RID=777H9BWJ013) |
| *Danio rerio* | TRIM2 | [NP_001014393.1](http://www.ncbi.nlm.nih.gov/protein/62122935?report=genbank&log$=prottop&blast_rank=1&RID=7742PR32016) |
| *Danio rerio* | LAFORIN | XP_688154 |
|  |  |  |
| *Drosophila* | TRIM71 | [NP_524772.2](http://www.ncbi.nlm.nih.gov/protein/22026822?report=genbank&log$=prottop&blast_rank=3&RID=777Y9D0U011) |
|  |  |  |
| *C. elegans* | TRIM71 | [NP_001020999.1](http://www.ncbi.nlm.nih.gov/protein/71980716?report=genbank&log$=prottop&blast_rank=1&RID=777W2P3C011) |
|  |  |  |
| *N. vectensis* | TRIM71 | XP_001639778 |
| *N. vectensis* | LAFORIN | [XP_001624235.1](http://www.ncbi.nlm.nih.gov/protein/156357458?report=genbank&log$=prottop&blast_rank=1&RID=77A7PEZJ01R) |
|  |  |  |
| *B. floridae* | TRIM71 | [XP_002594088.1](http://www.ncbi.nlm.nih.gov/protein/260798200?report=genbank&log$=prottop&blast_rank=1&RID=777PU6SP013) |
| *B. floridae* | TRIM2 | [XP_002598292.1](http://www.ncbi.nlm.nih.gov/protein/260806841?report=genbank&log$=prottop&blast_rank=1&RID=7748F29C01S) |
| *B. floridae* | LAFORIN | [XP_002610182.1](http://www.ncbi.nlm.nih.gov/protein/260830467?report=genbank&log$=prottop&blast_rank=1&RID=77AZ8ZJ7013) |
|  |  |  |
| *C. intestinalis* | TRIM71 | [XP_002129130.1](http://www.ncbi.nlm.nih.gov/protein/198438213?report=genbank&log$=prottop&blast_rank=1&RID=777TTHNN01S) |
| *P. tetraurelia* | LAFORIN | [XP_001448925.1](http://www.ncbi.nlm.nih.gov/protein/145526228?report=genbank&log$=prottop&blast_rank=1&RID=77B5K75P011) |
| *T. termophila* | LAFORIN | [XP_001017661.1](http://www.ncbi.nlm.nih.gov/protein/118368910?report=genbank&log$=prottop&blast_rank=1&RID=77BC93B6013) |
| *E. tenella* | LAFORIN | Et_v1_Twnscn_Contig6817.tmp13 |
| *T. gondii* | LAFORIN | [XP_002367761.1](http://www.ncbi.nlm.nih.gov/protein/237836927?report=genbank&log$=prottop&blast_rank=1&RID=77AER714013) |
| *N. caninum* | LAFORIN | NC_LIV_081160 |
| *C. merolae* | LAFORIN | CMT465C |
|  |  |  |
| *A. carolinensis* | MALIN | ENSACAP00000003277 |
|  |  |  |
| *C. jacchus* | MALIN | XP_002746236 |
| *C. jacchus* | TRIM2 | XP_002745431 |
| *C. jacchus* | TRIM32 | XP_002806562 |
| *C. jacchus* | TRIM56 | XP_002744018 |
| *C. jacchus* | TRIM71 | XP_002807716 |
|  |  |  |
| *D. novemcinctus* | MALIN | ENSDNOP00000004736 |
|  |  |  |
| *E. europaeus* | MALIN | ENSEEUP00000005168 |
|  |  |  |
| *F. catus* | MALIN | ENSFCAP00000003931 |
|  |  |  |
| *G. aculeatus* | MALIN | ENSGACP00000011248 |
|  |  |  |
| *G. gorrilla* | MALIN | ENSGGOP00000016278 |
|  |  |  |
| *M. eugenii* | MALIN | : ENSMEUP00000013821 |
|  |  |  |
| *O. anatinus* | MALIN | XP_001508385 |
| *O. anatinus* | TRIM2 | XP_001514346 |
| *O. anatinus* | TRIM32 | XP_001508478 |
|  |  |  |
| *O. cuniculus* | MALIN | XP_002720908 |
| *O. cuniculus* | TRIM2 | XP_002716938 |
| *O. cuniculus* | TRIM32 | XP_002720557 |
| *O. cuniculus* | TRIM56 | XP_002722861 |
|  |  |  |
| *O. princeps* | MALIN | ENSOPRP00000003138 |
|  |  |  |
| *P. abelii* | MALIN | XP_002816509 |
| *P. abelii* | TRIM32 | XP_002820194 |
| *P. abelii* | TRIM56 | XP_002817834 |
|  |  |  |
| *P. capensis* | MALIN | ENSPCAP00000002293 |
|  |  |  |
| *P. vampyrus* | MALIN | ENSPVAP00000010207 |
|  |  |  |
| *T. rubripes* | MALIN | ENSTRUP00000037316 |
|  |  |  |
| *T. truncates* | MALIN | ENSTTRP00000006743 |
|  |  |  |
| *X. tropicalis* | MALIN | XP_002932735 |
| *X. tropicalis* | TRIM32 | NP_001096393 |
| *X. tropicalis* | TRIM71 | XP_002937892 |
|  |  |  |
| *H. magnipapillata* | TRIM71 | XP_002159489 |
